# A draft network of ligand–receptor-mediated multicellular signalling in human

**DOI:** 10.1038/ncomms8866

**Published:** 2015-07-22

**Authors:** Jordan A. Ramilowski, Tatyana Goldberg, Jayson Harshbarger, Edda Kloppman, Marina Lizio, Venkata P. Satagopam, Masayoshi Itoh, Hideya Kawaji, Piero Carninci, Burkhard Rost, Alistair R. R. Forrest

**Affiliations:** 1 RIKEN Center for Life Science Technologies, Division of Genomic Technologies, 1-7-22 Suehiro-cho, Tsurumi-ku, Yokohama 230-0045 Japan; 2 Department for Bioinformatics and Computational Biology-I12, Technische Universität München (TUM), Boltzmannstrasse 3, 85748 Garching, Germany; 3 TUM Graduate School, Center of Doctoral Studies in Informatics and its Applications (CeDoSIA), Boltzmannstrasse 11, 85748 Garching, Germany; 4 Luxembourg Centre for Systems Biomedicine, Campus Belval, 7 Avenue des Hauts Fourneaux, L-4362 Belval, Luxembourg; 5 RIKEN Preventive Medicine and Diagnosis Innovation Program, 2-1 Hirosawa, Wako, Saitama 351-0198, Japan; 6 Harry Perkins Institute of Medical Research, QEII Medical Centre and Centre for Medical Research, the University of Western Australia, PO Box 7214, 6 Verdun Street, Nedlands, Perth, Western Australia 6008, Australia

## Abstract

Cell-to-cell communication across multiple cell types and tissues strictly governs proper functioning of metazoans and extensively relies on interactions between secreted ligands and cell-surface receptors. Herein, we present the first large-scale map of cell-to-cell communication between 144 human primary cell types. We reveal that most cells express tens to hundreds of ligands and receptors to create a highly connected signalling network through multiple ligand–receptor paths. We also observe extensive autocrine signalling with approximately two-thirds of partners possibly interacting on the same cell type. We find that plasma membrane and secreted proteins have the highest cell-type specificity, they are evolutionarily younger than intracellular proteins, and that most receptors had evolved before their ligands. We provide an online tool to interactively query and visualize our networks and demonstrate how this tool can reveal novel cell-to-cell interactions with the prediction that mast cells signal to monoblastic lineages via the CSF1–CSF1R interacting pair.

## Abstract (web summary)

Cell-to-cell communication relies upon interactions between secreted ligands and cell surface receptors. Here, Ramilowski *et al.* present a draft cell-to-cell communication network based on expression of ligand-receptor pairs in 144 different human cell types.

## Graphical abstract

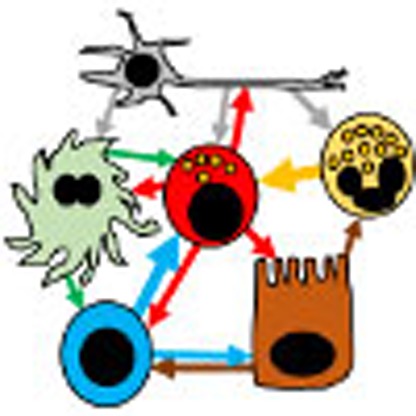

Development of multicellular organisms from unicellular ancestors is one of the most profound evolutionary events in the history of life on Earth^1^. In this transition, cells of multicellular organisms had to acquire various modes of cell-to-cell (intercellular) communication to develop and then control their coordinate functioning^2^. This process is critical during early embryonic development where the cell's differentiation and ultimate fate are controlled by communication with neighbouring cells^3^,^4^,^5^. In the developed organism, intercellular communication coordinates the activities of multiple cell types required for complex organismal processes such as immune response^6^, growth^7^ and homeostasis^8^. Defects in cell-to-cell communication, including dysregulation of autocrine signalling, are also medically important in cancer^9^, autoimmune^10^ and metabolic diseases^11^.

Despite its importance, studies of intercellular communication across specialized cells of higher metazoa have generally focused on communication between only a few cell types and via limited numbers of ligand–receptor pairs. Currently there are no reports of systematic studies trying to elucidate and quantify the repertoire of signalling routes between different cell types. To address this, we have systematically reviewed the expression profiles of 642 ligands and their 589 cognate receptors in our 1,894 literature-supported interacting pairs across a panel of 144 human primary cell types^12^. In particular, we used known interacting ligand–receptor pairs and public protein–protein interaction (PPI) information to generate the first large-scale draft map of primary cell-to-cell interactions. Highlighting their important role in the evolution of higher order metazoans, we show that receptors and ligands have more cell-type-specific expression profiles and are evolutionarily younger as a class than nuclear and cytoplasmic proteins. Applying a 10 tags per million (TPM; ∼3 transcripts per cell) detection threshold to our data, we find that primary cells express on average less than one-third of all ligands and receptors (roughly 140 ligands and 140 receptors). We also find that messages between any two given cell types are carried in a rather specific manner despite the hundreds of possible connecting paths and have significant potential for autocrine signalling. We also discuss in more detail the level of communication between different cell lineages. Finally, to benefit the research community, we provide an interactive visualization and query tool for ligand–receptor networks in humans (available at http://fantom.gsc.riken.jp/5/suppl/Ramilowski_et_al_2015/). This work is part of the FANTOM5 project. Data download, genomic tools and co-published manuscripts have been summarized at http://fantom.gsc.riken.jp/5/.

## Results

### PM and secreted proteins are young and cell-type specific

**Figure 1:**
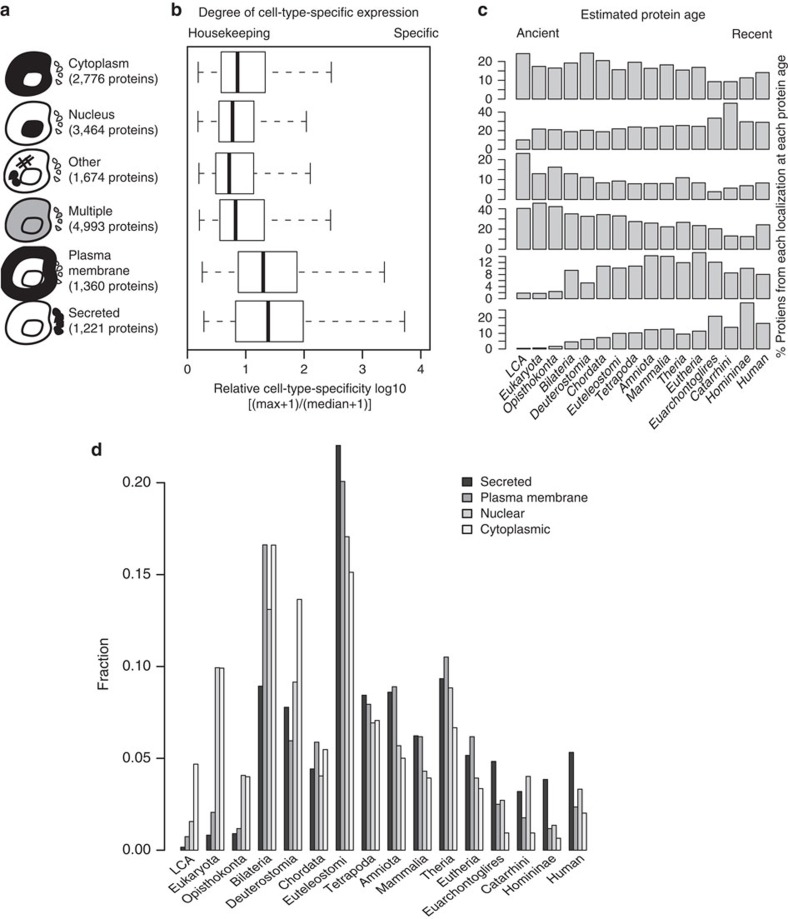
Relationship between protein subcellular localization, cell-type specificity and gene ages. (**a**) Breakdown of known subcellular localization of protein-coding genes expressed >1 TPM in at least one primary state for which protein ages were available. (**b**) Interquartile range distributions (whisker boxes) and relative cell-type specificity for each protein subcellular compartment from FANTOM5 primary cell expression profiles. Both secreted and plasma membrane proteins are significantly more cell-type specific than nuclear and cytoplasmic proteins (each Mann–Whitney *U*-test-adjusted *P* value<000.1). (**c**) Relative fractions of proteins at each evolutionary stage for selected subcellular localization (secreted, plasma membrane, nucleus, cytoplasmic and other) using the methods of Wagner^21^. All fractions at a given age add to 100%. (**d**) As in **c** but scaled for visualization purposes to the number of nuclear proteins. Both secreted (average age: 412.2 mya) and plasma membrane (average age: 517.2 mya) proteins are significantly younger than nuclear (average age: 663.1 mya) and cytoplasmic proteins (average age: 855.1 mya), each Mann–Whitney *U*-test-adjusted *P* value<000.1. Note: exact numbers of proteins for each subcellular localization class in each phylostrata are available in Supplementary Data 1.

Recently the FANTOM5 consortium used Cap Analysis of Gene Expression (CAGE) to generate a promoter level expression atlas^12^. Based on CAGE measurements across a collection of 975 human samples (primary cells, cell lines and tissues), gene expression profiles were classified as non-ubiquitous (cell-type restricted), ubiquitous-non-uniform and ubiquitous-uniform (housekeeping)^12^. Gene Ontology (GO)^13^ analysis of genes with cell-type-restricted expression showed their enrichment for proteins annotated with the terms receptor activity, plasma membrane (PM) and multicellular organismal process. This suggested that proteins involved in intercellular communication were more likely to have cell-type-restricted expression profiles. To explore this more systematically, we used protein experimental localization information^14^,^15^ and computational predictions^16^,^17^ (Methods) to classify human protein-coding genes (HGNC^18^ release 03 April 2014; http://www.genenames.org/cgi-bin/hgnc_downloads) based on the subcellular localization of the proteins they encode into: PM, secreted, cytosolic, nuclear, multiple and ‘other' proteins (Supplementary Data 1). Comparing the cell-type specificity of each class, we find that secreted and PM proteins are significantly more cell-type specific (Fig. 1) than proteins that localize to other cellular compartments (Mann–Whitney *U*-test, each adjusted *P* value<0.001). We also confirmed this trend using whole cell proteome data available for five haematopoietic primary cell types^19^ (Mann–Whitney *U*-test, each adjusted *P* value<0.001; Supplementary Fig. 1).

As cell-type-specific proteins are likely to appear with the emergence of new cell types and increased organismal complexity, we next examined the predicted ages of proteins from each subcellular localization using Protein Historian^20^ (pre-computed estimates based on Wagner parsimony^21^ and P-POD's^22^ OrthoMCL^23^ clustering of proteins in the PANTHER^24^ database were used). Evolutionary profiles of proteins from the different subcellular localizations show that secreted proteins (average age 412.2 mya) and PM proteins (average age 517.2 mya) are younger (Mann–Whitney *U*-test, each adjusted *P* values<0.001) than proteins that localize to the nucleus (average age 663.1 mya), cytoplasm (average age 855.1 mya) (Supplementary Data 1; Fig. 1c,d) or to other compartments. Using additional protein age estimates^25^,^26^, also confirmed the trend that PM and secreted proteins are generally the youngest proteins (Supplementary Fig. 2).

### Identification of putative ligand–receptor pairs

We next sought to examine in more detail PM and secreted proteins specifically involved in cell-to-cell communication. Building on previous efforts to curate lists of ligand–receptor pairs, we merged the lists from Database of Ligand−Receptor Partners (DLRP)^27^, IUPHAR^28^ and Human Plasma Membrane Receptome (HPMR)^29^ databases to generate a non-redundant set of 1,179 known interacting ligand–receptor pairs. Given that these resources originated many years ago, and are not extensively updated, we found many genuine ligand–receptor pairs were missing, for example GDF2->ACVR1 (ref. 30) and CCL4->CCR3 (ref. 31).

To extend this set, we first expanded the lists of candidate ligands and receptors by incorporating proteins predicted to be secreted and localized to the PM, respectively. We then searched for PPIs between all putative ligands and putative receptors (Supplementary Fig. 3a) as described in the Methods section. From this analysis, we inferred 2,117 experimentally supported interactions in the HPRD^15^ and STRING^32^ databases, which included 1,288 ligand–receptor pairs absent from our known collection of DLRP, IUHPAR and HPMR interactions.

From the above, we compiled a unique list of 2,467 known and inferred interactions. We next aimed to curate these interactions with a primary citation (PubMed ID), either by extracting the reference from the primary data sources (IUHPAR, HPMR and HPRD) or by manually searching the literature. Through the manual curation, we excluded 135 pairs, as the partners were not a genuine ligand or receptor, and found an additional 90 pairs. This resulted in a final curated set of 2,422 interactions: 1,894 interactions with primary literature support which we refer to as ‘reference' and use in our subsequent analysis, and the remaining set of 528 curated interactions without primary literature support we refer to as ‘putative' (Supplementary Fig. 3b). All ligand–receptor interactions are available in Supplementary Data 2.

### Receptors often evolved before their ligands

**Figure 2:**
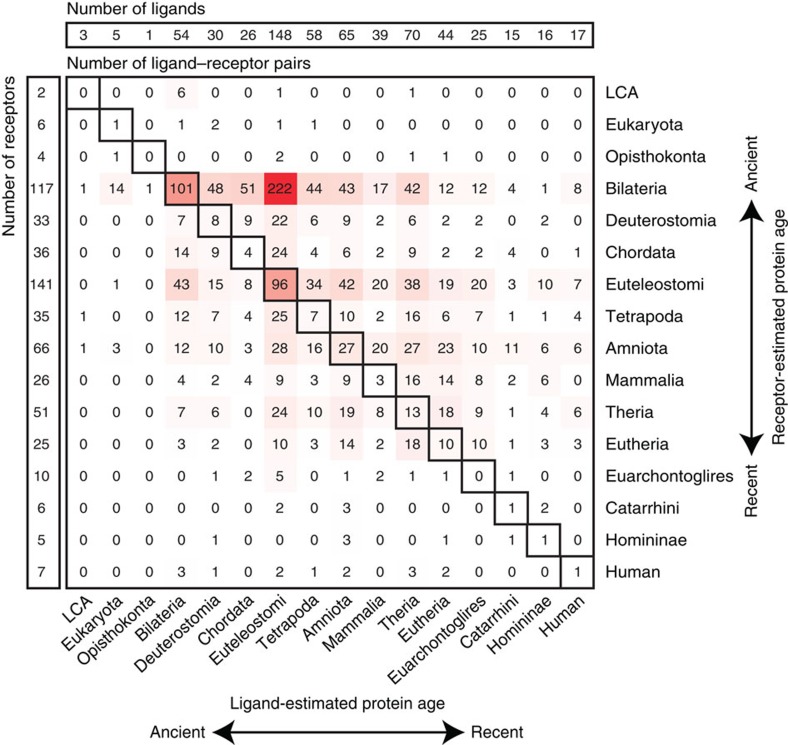
Comparative age of genes encoding receptors and ligands. Top and left panels list the number of ligands and receptors estimated to have arisen at each phylostratum using the method of Wagner^21^. Middle panel shows the number of ligand–receptor pairs observed in a given phylostrata. Intensity of red scales with the number of pairs. Note: many interactions (297 pairs) appeared at the same evolutionary stage (diagonal boxes), but we also observe a significant enrichment for 1,081 pairs where the receptor had appeared before the ligand as compared with 431 pairs, where the ligand had appeared first (binomial one-sided *P* value<0.001; 95% confidence interval [0.695, 1]).

Using our reference ligand–receptor pairs and the protein age estimates^20^,^21^, we examined whether the interacting partners appeared during the same evolutionary period as previously reported^33^, or if one had preceded the other^29^. We found that many cognate partners originated at the same phylostratum (273 pairs). However, we also observed an excess of 1,082 pairs where the ligand was younger than the receptor as compared with only 431 pairs where the ligand was older (Fig. 2). As ligands (median length 267 amino acids) are often shorter than receptors (median length 515 amino acids), we sought to exclude the possibility that length-related gene age estimate biases explain why ligands appeared to come after their cognate receptors. To address this, we generated a comparative matrix that consisted of interacting proteins extracted from HPRD (Supplementary Fig. 4), where one partner was shorter (lower quantile of all protein lengths) and the other was longer (upper quantile of all protein lengths). From this we found that in 1,933 out of 3,271 pairs the younger protein was shorter. Using a binomial one-sided test, adjusted for the length factor probability (1,933/3,271=0.591), we found that ligands are still significantly younger than their cognate receptors (*P* value<0.001; 95% confidence interval [0.695, 1]). We also confirmed the trend held with other measures of protein age^25^,^26^ (Supplementary Fig. 4c,d), and thus can conclude that for the majority of ligand–receptor pairs the ligands appeared after their cognate receptors.

### Receptor and ligand repertoires of mammalian cell types

To reliably determine the repertoire of ligands and receptors in each primary cell type using CAGE data requires extracting their expression levels at a certain detection threshold. In FANTOM5, we previously used 10 TPM as a conservative detection threshold as it theoretically equates to ∼3 transcript copies per cell^34^. Cell-to-cell signalling, however, requires that these transcripts are translated into proteins, therefore we examined the level of protein support at three different thresholds of CAGE expression levels (10, 50 and 100 TPM). For the comparison, we used B lymphocytes as they have been extensively studied over the past 50 years, have large amounts of flow cytometry data available and their whole cell proteome was recently measured by Kim *et al.*^19^. At the 10 TPM threshold, 82% (147/179) of the ligands and receptors detected by CAGE were also found in the whole B-cell proteome data set or were previously reported as detectable in B cells by antibody staining (Supplementary Data 3). At the higher thresholds, the level of support increased; (82/83—99%) and (57/57—100%) ligands and receptors detected by CAGE at 50 and 100 TPM, respectively, were found in the proteome data, but many true positives were lost. In addition, to estimate the fraction of potential false negatives at 10 TPM, we compared the set of gene products not detected in the FANTOM5 B-cell transcriptome but present in the proteome data of Kim *et al.*^19^ to a high quality microarray data set collected for the Haematlas project^35^. We found that only 4% of these gene products (8/192 with unique probes on the arrays) had detectable transcripts, in contrast to 78% of gene products detected by FANTOM5 at 10 TPM (125/161 with unique probes on the arrays). We conclude that the remaining 184 proteins detected in the proteome data only, are most likely not produced by B cells but instead are either false positives of the proteome analysis or non-cell autonomous^36^ contributions to the proteome. In particular, we note that well known liver specific proteins ASHG, ALB and APOB and the testis-specific AMH were detected in the B-cell proteome yet there is no evidence of their expression in any other B-cell transcriptome data set (not restricted to FANTOM5 and Haematlas). We thus concluded that applying the 10 TPM detection threshold is likely to yield relatively low false positive and false negative rates and used it for the remainder of the manuscript.

Systematically examining ligand and receptor expression at this threshold across 144 primary cell types, we detected 464 ligands and 477 receptors expressed in at least one cell type (376 ligands, 369 receptors at 50 TPM, 309 ligands and 286 receptors at 100 TPM). We also observed that on average, each cell type expresses ∼30% of these genes (∼140 ligands and ∼140 receptors), (82 ligands and 60 receptors at 50 TPM; 59 ligands and 35 receptors at 100 TPM).

Next we carried out hierarchical clustering of the receptor and ligand expression patterns across the primary cell types (Supplementary Fig. 5). We found that most cell types largely clustered by cell lineage and shared sets of lineage-specific receptors and ligands. For example, we identified a cluster of ligands and receptors that are enriched in all endothelial cell types, which included two of the vascular endothelial growth factor receptors *KDR* and *FLT4*. We also highlight a vascular smooth muscle cell cluster with a striking enrichment for cytokines and chemokines (*CXCL1, CXCL3, CXCL5, CXCL6, CXCL11, IL6, IL11, CCL7, CCL8, GDF6, BMP2, NPPB* and *CSF3*). The expression profiles for all ligands and receptors found in reference and putative interaction sets across the 144 primary cells are available in Supplementary Data 4.

### General statistics of the cell-to-cell signalling network

**Figure 3:**
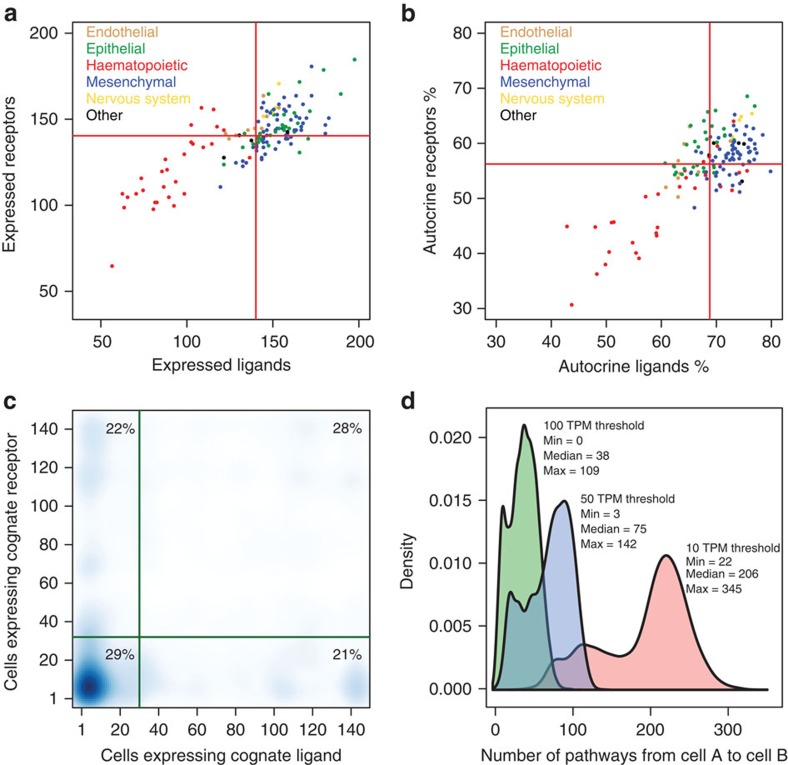
Summary statistics of ligand and receptor usage in human primary cells. (**a**,**b**) Each data-point corresponds to a primary cell type. Colours indicate broad lineage classes. (**a**) Number of ligands (*x*-axis) versus numbers of receptors (*y*-axis) expressed in each cell type. (**b**) Autocrine signalling in primary cell types. *X*-axis shows the fraction of ligands expressed by a given cell where the receptor is also expressed on the same cell. *Y*-axis shows the reciprocal for the fraction of receptors on a given cell where the ligand is also expressed. The red lines in **a**,**b** show the mean numbers of ligands or receptors in each plot. (**c**) Density plot showing the number of cells in which each cognate ligand–receptor pair is expressed. Medians are shown as green lines. For all plots in **a**–**c** a threshold of 10 TPM was used. (**d**) Distribution of the number of possible ligand–receptor paths between ligand-secreting cell A and receptor-expressing cell B calculated for all 144 × 144 possible cell-pair permutations across 10, 50 and 100 TPM CAGE detection thresholds.

Broadly classifying the cell types using cell ontologies^37^ into endothelial, epithelial, haematopoietic, mesenchymal, nervous system and other lineages, and reviewing their ligand/receptor expression profiles, we observed that the blood lineages appeared to be outliers in that they express less ligands on average (∼92, ∼51, ∼36 ligands at 10, 50, 100 TPM, respectively; Mann–Whitney *U*-test *P* values<0.001) and less receptors on average (∼120 receptors at 10 TPM; Mann–Whitney *U*-test *P* value<0.001) compared with the other lineages (Fig. 3a, Supplementary Fig. 6a,b). This suggests that immune cells use fewer paths to broadcast their state to their neighbours. We also observe that on average two-thirds of ligands and receptors expressed from any given cell can potentially bind to at least one of its cognate partners on the same cell type (Fig. 3b), indicating that the extent of autocrine signalling is significant.

Based on the expression profiles of ligands and receptors across the panel of 144 primary cells, we then considered specificity of expression of 1,287 interacting ligand–receptor pairs (Fig. 3c). The median number of cell types that express any given ligand was 30, while the median number of cell types that express any given receptor was 32 (threshold of ≥10 TPM). Using these medians to classify genes as specific or broad, we found that 29% of all pairs have cell-type-restricted expression of both their ligand and receptor, 43% had restricted expression of only the ligand or the receptor and 28% of pairs used both broadly expressed ligands and broadly expressed receptors. Thus 72% of pairs involve at least one partner with restricted expression, facilitating selective information transfer via the use of restricted transmitters and/or receivers. Further examining our complete set of 1,287 ligand–receptor signalling paths between all cell types, we found that at a threshold of 10 TPM for both interacting partners all 144 cell types had the potential to signal to each other through a minimum of 22 signalling paths and that on average a pair of cells can communicate using 190 of these paths (Fig. 3d). Only at a threshold of 100 TPM did we predict some cell pairs would not communicate. Repeating the analyses of Fig. 3a–c at the 50 and 100 TPM thresholds reduced the number of detected pairs, but most findings were on a similar scale (Supplementary Fig. 6).

To understand the biology of ligand–receptor pairs that use restricted or broadly expressed transmitters/receivers, we used the DAVID^38^ tool (http://david.abcc.ncifcrf.gov/) to search for enrichment of protein domain, molecular function and biological process annotations in the quadrants of Fig. 3c. Pairs involving broadly expressed receptors and ligands were enriched for EGF domains, integrin binding and blood vessel development terms. Pairs with broadly expressed ligands but restricted receptor expression were enriched for G protein coupled receptor, protein kinase domains and chemokine, receptor kinase, cyclic nucleotide and second messenger signalling terms. Pairs involving restricted ligands and broadly expressed receptors were enriched for transforming growth factor-beta domains, growth factor activity and regulation of protein phosphorylation/modification terms. Finally, pairs involving restricted ligands and restricted receptors were enriched for small chemokine, c-type lectin- and rhodoposin-like domains and peptide receptor, cytokine, cell-to-cell signalling and locomotory behaviour terms (Supplementary Data 5).

### Ligand–receptor signalling network interface

**Figure 4:**
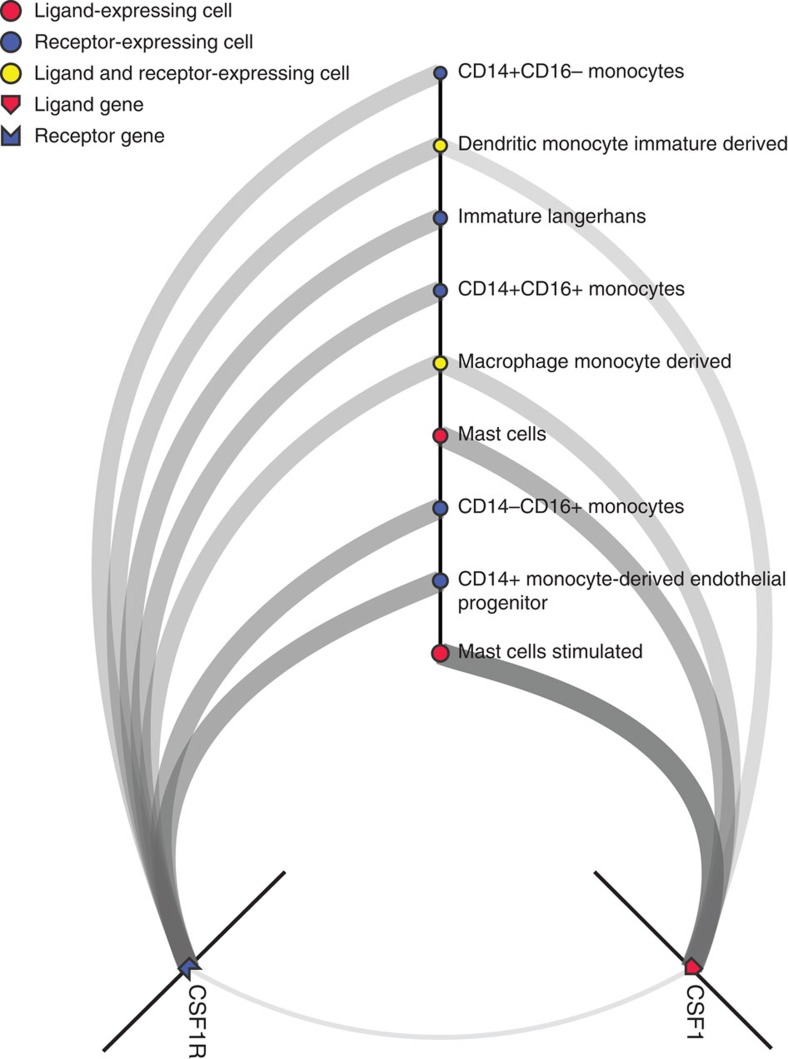
Ligand–receptor signalling network interface (hive view). The results of a search for the CSF1–CSF1R ligand–receptor pair, filtered for the top cell-to-cell paths (ranked by the product of CSF1 and CSF1R expression). In this network, stimulated mast cells express the highest levels of CSF1 (1,109 TPM), while CD14+ derived endothelial progenitor cells express the highest levels of CSF1R (699 TPM). Users can select cells and/or ligand–receptor (LR) pairs of interest and filter edges and nodes based on expression levels of L and R. The interface is available at: http://fantom.gsc.riken.jp/5/suppl/Ramilowski_et_al_2015/.

Using the ligand and receptor pairs described above, we then calculated all cell-to-cell edges where both ligand and receptor were expressed in at least one primary cell state (≥10 TPM). To benefit the research community, we provide an online resource that visualizes on demand cell-to-cell networks for any given ligand–receptor pair across all 144 primary cell types. The tool allows users to select primary cells and ligand–receptor pairs to be visualized, and then filters the edges (receptor expression × ligand expression) and nodes (cells) based on the expression levels. Visualized networks can be downloaded as SVG (scalable vector graphics) or in a data format compatible with other network visualization platforms such as Cytoscape^39^ and Gephi^40^ for additional exploration. In Fig. 4, we show an example of top cells communicating via the CSF1 ligand–CSF1R receptor pair, where mast cells are the major broadcasters (the highest levels of CSF1 expression), and monocytes and related cells are the major recipients (the highest levels of CSF1R expression) of these signals. We also note that monocyte-derived macrophages demonstrate autocrine signalling via this pair, expressing both CSF1 and CSF1R at notable levels. Additional use cases are provided in Supplementary Note 1.

### Multicellular processes in cell-to-cell communication

**Figure 5:**
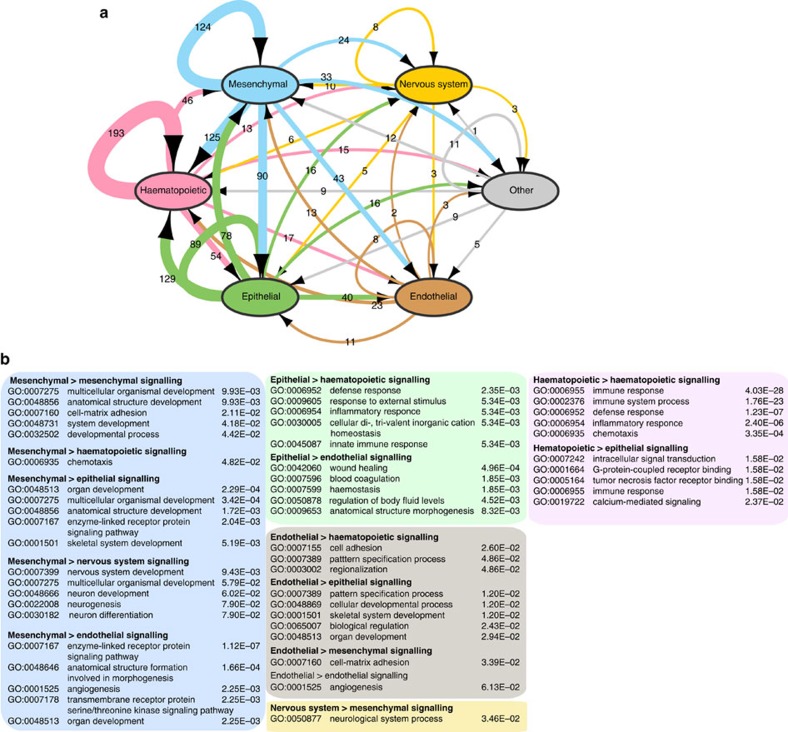
Enrichment of multicellular processes in the max-signalling pair network. About 1287 ligand–receptor (LR) pairs where the receptor (R) and the ligand (L) are expressed above 10 TPM in at least 1 primary cell state are considered. For each LR pair, the cell expressing the highest level of L and highest level of R are considered the major-signalling pair. The number of major-signalling pairs for all LR are then counted for all cell types profiled and summarized into intra- and inter-lineage signalling. (**a**) Summary network showing the level of signalling across and within lineages. Edges are scaled and numbered with the number of pairs between broadcasting and target cell. (**b**) Gene Ontology enrichment analysis of receptors and ligands involved in signalling between different lineages. The background gene set was the full set of receptors and ligands shown in **a**, and the test sets are the genes from the pairs shown on the edges. Only the top five biological processes with at least five enriched genes and their Benjamini-corrected *P* values are shown. Number of cell types considered in each lineage are: mesenchymal (63), nervous system (4), other (5), endothelial (9), epithelial (34) and haematopoietic (29).

Conceptually, our entire cell-to-cell communication network can be thought of as multi-edge (tens to hundreds of paths between any two cells), weighted (variable ligand/receptor expression levels), directed (cell A signals to cell B), hypergraph (a ligand can be secreted from multiple cells to interact with its cognate receptor(s) on multiple cells) network with millions of potential connections. To reduce the complexity of this graph (namely to remove its hypergraph aspect), we extracted the pair of cells that expressed the highest level of ligand and the highest level of receptor; we refer to these as the major-transmitter and major-receiver, respectively, and to the pair as the major-signalling pair (Supplementary Data 6; these major-signalling pairs are likely to be of the highest physiological significance). Using the six cell lineage classes described above, that is, endothelial, epithelial, haematopoietic, mesenchymal, nervous system and other lineages, we counted the number of major-signalling pairs that were communicating within and across lineages (summarized in Fig. 5). As the numbers of cell types in each lineage varied, we tested whether the number of ligands and receptors that were found at maximum levels in a given lineage were different than expected by chance. We observed that although the mesenchymal lineages had more cell types (63) (cf. epithelial (34) and haematopoietic (29)), they had significantly less ligands and receptors than expected by chance (false discovert rate (FDR)-corrected binomial *P* values<0.001 for both ligands and receptors). Conversely, the blood lineages were significantly more often expressing the maximum levels of various ligands and receptors than expected (FDR-corrected binomial *P* values<0.001 for both ligands and receptors). Similarly, we noticed that epithelial and nervous lineages were significantly more often expressing the maximum levels of various receptors and ligands than expected (FDR-corrected binomial *P* values<0.001). For detailed results of this analysis, see Supplementary Data 7 and Supplementary Fig. 7.

Next, given the distribution of max-receivers and max-transmitters across and within the lineages (and now ignoring the numbers of cell types in each lineage class), we checked whether any paths (cell-lineage-A to cell-lineage-B) were more common than expected by chance. We observed a striking enrichment for intra-lineage signalling for cells in the haematopoietic, mesenchymal and nervous system lineages, where both receptors and ligands were more likely to be bound by interacting partners from cells of the same lineage (FDR-corrected binomial *P* values<0.001). In contrast, we did not observe such significant enrichment in any inter-lineage signalling (FDR-corrected binomial *P* values>0.2; Supplementary Data 7).

We next carried out GO enrichment analysis on the pairs of genes used for communication between or within lineages using the entire set of receptors and ligands (Supplementary Data 6) as the background to avoid enrichment of generic terms such as PM and secreted. As might be expected, genes involved in intra-haematopoietic lineage signalling were enriched for immune, defense and inflammatory response genes, whereas genes involved in intra-endothelial lineage signalling were involved in angiogenesis. Inter-lineage signalling revealed some of the most interesting sets of genes enriched in processes that are known to require the concerted actions of cells from multiple lineages. Mesenchymal cell signalling to haematopoietic, nervous system and endothelial cells, respectively, revealed relevant processes such as chemotaxis; nervous system development, neurogenesis and neuron differentiation; and angiogenesis, respectively. Similarly epithelial to haematopoietic signalling was enriched for genes involved in defense response, inflammatory response and innate immune response, while epithelial to endothelial signalling was enriched for genes involved in wound healing, blood coagulation and haemostasis (see Supplementary Data 6 for the full set of enriched terms). Notably, examining signals to haematopoietic lineages from three different lineages, mesenchymal, epithelial and haematopoietic cells, revealed different biological processes. Mesenchymal to haematopoietic signals were enriched for proteins annotated with the term chemotaxis, epithelial to haematopoietic signals were enriched with the term defense response and haematopoietic to haematopoietic signals was most highly enriched for the term immune response. These results reflect that distinct multicellular processes are at work (even when one of the cellular partners is the same; haematopoietic) and that only by considering pairs in this way can they be revealed.

## Discussion

To date there is little systematic literature on the degree of intercellular communication between human cell types. The most comprehensive collections of literature-derived ligands and receptors are the DLRP^27^ and the HPMR^29^, however, neither of these address the complex network of signals between normal cell types. We have compiled and largely expanded the set of 1,179 known ligand–receptor pairs to 1,894 primary literature-supported and 528 putative (interacting PM and secreted proteins) pairs. Using these ligand–receptor pairs and the unique FANTOM5 resource, which provides expression levels of these genes in the major human primary cell types, we have constructed and analysed the first large-scale map of cell-to-cell communication and revealed extensive intra- and inter-lineage signalling.

Based on expression profiles of proteins classified into different subcellular localization classes, we found, as might be expected, that secreted and PM proteins have the most cell-type-specific expression profiles. Using different gene estimates for these proteins, we observed that younger proteins are also more likely to be PM or secreted proteins, while older ones are more likely to be nuclear or cytoplasmic. Overall this suggests that as metazoans continued to evolve new cell types, new cell-type-specific PM proteins were required to specifically tag these new cell types and that new secreted proteins were required to report the state of the new cell type to other cells, these are key features required for specific cell-to-cell communication. Examining the evolutionary appearance of interacting ligand and receptor pairs with the method of Wagner^21^, we observe a burst of new receptors and ligands appearing after Opisthokonta at Bilateria and Euteleostomi, however, we also consistently observe, using various gene estimate methods, a general bias for receptors to appear before their cognate ligands. This would seem to fit with one of the models for ligand–receptor pair formation proposed by Ben-Shlomo *et al.*^29^, where existing PM proteins (pre-receptors) adopt ligands that modulate their activity.

To benefit the research community, we have created a web tool (http://fantom.gsc.riken.jp/5/suppl/Ramilowski_et_al_2015/vis) that allows users to find the following: (i) the most highly expressed receptors and ligands for any cell type of interest; (ii) the most specific signalling paths between any two cell types and (iii) all cells that use a defined set of ligand–receptor pairs (Supplementary Note 1). For known pairs, we provide links to the primary literature via PubMed, but also allow the user to examine putative novel pairs identified by our study. We suspect that many of these putative pairs are genuine based on known interactions of paralogues (for example, ENG is known to be bound by INHBA, but we also predict binding of the paralogue INHBE; similarly CCR9 is known to bind to CCL25 but we predict it also binds CCL13)^41^,^42^. In addition, the genes in some of these putative pairs are co-implicated in disease, for example, APOE is predicted as a ligand for CHRNA4 and several papers have shown a genetic interaction between these genes affecting age-related cognitive decline^43^ and white matter volume^44^; similarly BDNF is predicted as a new ligand for DRD4 and a genetic interaction between these two genes has been found associated with bulimia nervosa^45^.

The network of connections between cells appears to be incredibly complex with many routes between the same two cells at different levels of expression and specificity. Unlike a transcriptional regulatory network, which is generally simplified to a set of genes as nodes and transcription factor binding as regulatory edges, a cell-to-cell network consists of cells as nodes and between any two cells there can be hundreds of potential messages passed between them. In addition, it is not easy to model the physiological response of the node (the cell) without extensive biochemical data. Herein, focusing only on the major-signalling pairs (the pair of cells that expressed the highest level of ligand and highest level of receptor for each interacting pair) and abstracting the network further, grouping cells into lineages (Fig. 5) we showed a significant bias for intra-lineage communication. In particular for blood, more than half of the ligands were targeted to other blood cells. GO enrichment analysis on the pairs of genes used in communicating, within or between lineages, showed that genes involved in intra-haematopoietic lineage signalling were enriched for immune response and inflammation genes, whereas genes involved in intra-endothelial lineage signalling were involved in angiogenesis. Signalling of the mesenchymal and epithelial lineages to haematopoietic cells was enriched for chemotaxis and defense response terms, respectively.

Examining individual edges in more detail, we found examples of lineage-specific paralogues being used to communicate with ligand–receptor families that are often thought of as restricted to another lineage. For example, chemokines and their receptors are generally thought of as haematopoietic; however, we find chemokines that are most highly expressed in mesenchymal, epithelial and endothelial lineages and appear to be used for communication to haematopoietic lineages. Signalling from mesenchymal to haematopoietic cells, we find CCL11 and CXCL12 chemokines. CCL11 is highly expressed in smooth muscle cells, in particular non-vascular tissues (colonic, oesophageal, prostatic and uterine), and can bind to the CCR3 receptor expressed on myeloid cells. This association has functional evidence as CCL11 expression in uterine smooth muscle cells has been implicated in the recruitment of mast cells via CCR3 into uterine cellular leiomyosarcoma^46^ and with eosinophilic infiltration of other tissues in disease^47^. Similarly, we find that CXCL12 (which binds to CD4, CXCR3 and CXCR4 on haematopoietic cells) is highly expressed in synoviocytes. CXCL12 has been shown to be upregulated in rheumatoid arthritis synoviocytes and influences T-cell accumulation in the disease^48^. We also observe epithelial to haematopoietic signalling via CCL15 binding to CCR1/3 and via CCL16 binding to CCR1/2/5/8 and HRH4, and endothelial to haematopoietic signalling via CCL14 binding to CCR1/3/5. In the case of CCL16, this ligand is most highly expressed in hepatocytes^49^, is a trigger effector for macrophages via CCR1 (ref. 50), and recruits eosinophils via the non-canonical receptor HRH4 (ref. 51).

Since the wealth of observed paths between cells of interest is too large to go into additional detailed examples here, we direct the user to the web tool to explore further. Systematic examination of ligand and receptor expression across 144 primary cell types can, however, give insights enabling us to make some general observations. Most cells express on the order of 140 receptors and 140 ligands at appreciable levels, equating to roughly 30% of all ligands and receptors, with the exception of haematopoietic cells, which express only 18–22% of all ligands and receptors on average. This suggests that they use fewer paths to broadcast their state to their neighbours, but given the large number of haematopoietic cells acting as major receivers or transmitters as seen in Fig. 5 this may also reflect greater specificity in the set of cells they target. Another observation was that on average 70% of ligands expressed by any given cell type can bind a cognate receptor on the same cell type, and conversely 60% of receptors expressed by a cell can bind ligands expressed by the same cell type. This may indicate that many autocrine signalling paths are used to reinforce the cell state, or that juxtacrine signalling to cells of the same type is used to communicate the state to its neighbours. Examining the numbers of cell types expressing each ligand and receptor, we find that 72% of pairs have at least one partner (ligand or receptor) with restricted expression, which further suggests the importance of ligand–receptor cell-type expression specificity for selective information transfer in multicellular organisms.

We acknowledge that there are several simplifications and assumptions that we made in our analyses. We use CAGE to measure mRNA levels, but physiologically meaningful interactions of endogenous ligands and receptors require that they are expressed as proteins, correctly post-translationally modified and then localized to the PM or extracellular space. Without PM and secretome proteomics data on human primary cells^19^,^52^, transcriptomics data is our best alternative, and defendable given the good degree of correlation between mRNA and protein levels^52^. We must note, however, that the analysis of whole cell proteomics is not as mature as the transcriptome analyses. While 82% of the ligands and receptors detected by CAGE in B cells also had protein level support, our literature review found that many of the proteins detected only in the B-cell proteome of Kim *et al.*^19^ (and not detected in the FANTOM5 B-cell transcriptome) are most likely not produced by B cells and are likely to be false positives of the analysis or non-cell autonomous^36^ contributions to the proteome.

In addition, we do not consider direct cell-to-cell contact, which is particularly important in juxtacrine signalling. We assume that binding elicits some state change in the target cell, yet to correctly estimate physiological responses, affinity of ligands, receptor internalization, recycling, intracellular signalling pathways and whether the receptor requires to dimerize or interact with additional proteins would need to be considered. We are not aware of comprehensive data covering these aspects across primary cell types and have thus abstracted to the simple requirements that the receptor and ligand are expressed and known to bind. We also recognize that we need to add new cell types to the resource over time as new CAGE and RNA-seq data sets become available. This is necessary as 177 ligands and 112 receptors were not expressed at appreciable levels in the 144 primary cell types considered. In particular, GO analyses revealed that the missing proteins were often involved in neuropeptide signalling, virus response (especially alpha interferons) or were hormones expressed in very restricted cell populations (for example, insulin from beta cells, gastrin from G cells and gonadotropin-releasing hormone 1 from GnRH neurons) (Supplementary Data 8).

Despite these caveats, we recover known and discover novel physiologically important cell-to-cell relationships including the CSF1–CSF1R network (Fig. 4). CSF1 is a key growth factor for macrophages and CSF1R is expressed on most myeloid lineage cells^53^. As previously reported, we observe an autocrine signalling potential of monocyte-derived macrophages^54^, but also for immature monocyte-derived dendritic cells and basophils. Most interestingly, we observed that mast cells produce the highest levels of CSF1 and upregulate it on stimulation. To our knowledge this is a novel relationship revealed by our analysis.

In summary, we introduce the first large-scale map of cell-to-cell signalling by presenting a network, where cells are the nodes and receptor–ligand pairs form the edges. This information is critical for organism-level systems biology (molecular physiology) to better understand the cellular participants and signalling pairs used in complex cellular networks employed in disease, development, immune response and normal homeostasis. Finally, at an immediate and practical level it will allow us to find novel factors for improved culture of various cell types, as we have shown recently with the use of BMPs for mast cells^55^ and CCL2 for embryonic stem cells^56^. In the future, we hope to cover more primary cell types by incorporating single cell expression data sets^57^ including those that capture spatial relationships^58^ and allow us to examine juxtacrine signalling between neighbouring cells.

## Methods

### Reference set of human protein-coding genes

We downloaded the set of 19,074 HGNC^18^ protein-coding genes (03 April 2014) and used the subset of 19,053 genes with an existing UniProt ID for our analyses (Supplementary Data 1). HGNC-approved symbols were used as the common identifier throughout our analyses to match identifiers from other data sources.

### FANTOM5 protein-coding gene expression profiles

The expression profiles of protein-coding genes in primary cells were obtained from the FANTOM5 promoterome expression atlas^12^ (403 samples corresponding to 144 primary cell types—Supplementary Data 9). Expression of each gene across a given primary cell was estimated from the summed expression of its promoters across each library and then averaged for biological and/or technical replicates (most libraries are biological triplicates). The summarized gene expression data is available at http://fantom.gsc.riken.jp/5/suppl/Ramilowski_et_al_2015/data/ as ‘ExpressionGenes.txt'.

### Subcellular localization classifications

For each protein-coding gene, we first extracted known subcellular localization annotations from the UniProtKB and from the HPRD^15^. Over one-third of these proteins lacked experimental localization information, therefore we used the computational tools LocTree3 (ref. 16) and PolyPhobius^17^ to predict subcellular localizations and transmembrane helices (TMHs) for all proteins in our data set. Predictions were run on protein sequences of the Reference Human Proteome (http://www.ebi.ac.uk/reference_proteomes) from the European Bioinformatics Institute, and if not available we used the longest isoform sequence from UniProt (ftp://ftp.uniprot.org/pub/databases/uniprot/current_release/knowledgebase/proteomes/).

Tier1 (12,976 proteins with known localizations): the subcellular localization of the protein is already annotated in UniProt or HPRD. From UniProt, we accept all experimentally verified and probable subcellular localizations. From HPRD, we accept all localizations with associated PubMed ID. For PM annotations from HPRD, we additionally require that at least one TMH is predicted for this protein by PolyPhobius. Tier2 (5,906 proteins): The remaining proteins were annotated using the subcellular localization predicted by LocTree3. Here we also required at least one TMH predicted by PolyPhobius for PM proteins and at most one TMH predicted for secreted proteins. The proteins that did not meet the last criteria could not be classified and were denoted as ‘n/a' (171 proteins).

For the analysis purposes, we excluded these unclassifiable proteins and assigned the others into one of the six localization classes: cytoplasm, multiple, nucleus, other, PM and secreted.

### Known ligand–receptor interactions

Known ligand–receptor pairs were downloaded from the DLRP^27^ (http://dip.doe-mbi.ucla.edu/dip/dlrp/dlrp.txt), IUPHAR^28^ (http://www.guidetopharmacology.org/ DATA/interactions.csv) and HPMR^29^ (http://receptome.stanford.edu/) databases (download dates 23 July 2013, 23 June 2014 and 03 July 2014, respectively). After mapping to current HGNC symbols, we obtained 469, 371 and 855 ligand–receptor pairs from DLRP, IUPHAR and HPMR, respectively.

An additional 128 orphan ligands and 479 orphan receptors were also downloaded from HPMR (26 June 2014).

### Prediction of novel ligand–receptor pairs

Computationally inferred ligand–receptor pairs (2,117) were obtained by searching for experimentally validated PPIs (HPRD— http://www.hprd.org/download and STRING^32^— http://string.uzh.ch/download/protected/string_9_1/protein.links.full.v9.1/9606.protein.links.full.v9.1.txt.gz databases) between a set of putative ligands and putative receptors (Supplementary Fig. 3a). Putative ligands (2,132) were compiled from known interacting ligands (470), orphan HPMR ligands (140) and from our set of secreted proteins that were not found in the set of known receptors (1,866). Putative receptors (2,363) were compiled from known interacting receptors (448), orphan HPMR receptors (488) and from our set of PM proteins that were not found in the set of known ligands (2,076).

From HPRD (Release9_062910), we obtained 1,322 binary PPIs supported by any of the three types of evidence source (*in vitro*, *in vivo* and yeast 2-hybrid). In STRING9.1, we found 1,362 ‘*Homo sapiens*' physical-binding interactions (confidence score ≥700) and 428 experimental interactions (confidence score ≥700). STRING's internal ‘ENSP IDs' were first matched using Ensembl BioMart mapping of ‘Ensembl Protein ID' to ‘HGNC Gene Symbol' for *Homo sapiens* genes (GRCh37.p13) then further matched to a current HGNC ‘Gene Symbol'.

### Protein age estimates

Pre-computed protein age estimates were downloaded from Protein Historian: Protein Age Estimation and Enrichment Analysis tool^20^ (http://lighthouse.ucsf.edu/ProteinHistorian/downloads.html) and from the phylostratigraphic age estimates for the human loci as described by Neme *et al*.^26^ Protein historian phylogenetic age estimates relied on the P-POD^22^ (Princeton Protein Orthology Database), and were based on an OrthoMCL^23^ clustering of all proteins in the 48 species present in v7.0 of the PANTHER^24^ (Protein analysis through evolutionary relationships) classification system. They used either Wagner^21^ or Dollo^25^ parsimony ancestral reconstruction algorithms.

### Statistical analysis

Mann–Whitney *U*-tests for subcellular localizations specificity, age comparisons and distribution of ligands/receptors in blood versus all others were carried out using R package wilcox.test with default parameters. Binomial tests for ligand–receptors pairs age comparisons, for lineage-specific over- and under-representation of ligands/receptor in the major-signalling pairs and for the bias in cell-to-cell intra- and inter-lineage signalling were carried out using R package binom.test with default parameters. When necessary, *P* values were corrected using R package p.adjust with p.adjust.method=‘fdr'.

### GO and InterPro domain enrichment analysis

GO and InterPro^59^ enrichment analysis for ligands and receptors pairs in Fig. 3c were carried out using the DAVID^38^ tool. All HGNC identifiers were first converted to Entrez GeneIDs. GO analysis in Fig. 5 was carried out using GOstat^60^ (http://gostat.wehi.edu.au/). Lists of background and foreground Entrez GeneID sets are included in Supplementary Data 5 and 6.

### Online visualization resource

The interactive visualization and query tool for ligand–receptor networks was developed using custom and open source tools. The vector graphic visualization was generated using the D3.js visualization library^61^ (http://d3js.org/). The application interface was developed using the AngularJS web application framework (https://angularjs.org/) and the twitter bootstrap front-end framework (http://getbootstrap.com/).

The visualization interface takes the expression files generated in this study along with other metadata in tabular format to generate the network/hive visualization as shown in Fig. 5. An online version of the resource is located at: http://fantom.gsc.riken.jp/5/suppl/Ramilowski_et_al_2015/vis/ and mirrored at http://forrest-lab.github.io/connectome. The source code is under MIT license and is available at: https://github.com/Hypercubed/connectome/ (version: /tree/v0.1.0).

## Additional information

**How to cite this article:** Ramilowski, J. A. *et al.* A draft network of ligand–receptor-mediated multicellular signalling in human. *Nat. Commun.* 6:7866 doi: 10.1038/ncomms8866 (2015).

## Supplementary Material

Supplementary Figures and Supplementary Note Supplementary Figures 1-18 and Supplementary Note 1

Supplementary Data 1 Consensus subcellular localization and ages of all proteins encoded in the human genome

Supplementary Data 2 Curated and putative ligand-receptor pairs in human.

Supplementary Data 3 Comparison of receptors and ligands detected in B cells by CAGE with protein evidence (proteome and literature).

Supplementary Data 4 Ligand and receptor repertoires of 144 primary cell types.

Supplementary Data 5 InterPro protein domain and Gene Ontology enrichment of receptors and ligands that are broadly or restrictedly expressed.

Supplementary Data 6 Major signalling pairs of cells based on maximum receptor and ligand expression values and extended Gene Ontology enrichment analysis results.

Supplementary Data 7 Testing for bias in the sets of cells expressing the highest levels of receptors and ligands (i.e. Max-transmitter, max-receiver) and bias in signalling from cell type A to cell type B.

Supplementary Data 8 Receptor and ligands not detected in the 144 primary cell types and Gene Ontology enrichment analysis results.

Supplementary Data 9 List of cell types and FANTOM5 CAGE libraries used in this study.
