# Corrigendum: A draft network of ligand-receptor-mediated multicellular signalling in human

**DOI:** 10.1038/ncomms10706

**Published:** 2016-02-10

**Authors:** Jordan A. Ramilowski, Tatyana Goldberg, Jayson Harshbarger, Edda Kloppmann, Marina Lizio, Venkata P. Satagopam, Masayoshi Itoh, Hideya Kawaji, Piero Carninci, Burkhard Rost, Alistair R. R. Forrest

Nature Communications 6: Article number: 786610.1038/ncomms8866 (2015); Published: 07222015; Updated: 02102016.

The original version of this Article contained an error in the spelling of the author Edda Kloppmann, which was incorrectly given as Edda Kloppman. This has now been corrected in both the PDF and HTML versions of the Article.

