# Supplementary material for: A draft network of ligand–receptor-mediated multicellular signalling in human
Source: Nat Commun. 2015 Jul 22;6:7866. doi: 10.1038/ncomms8866 (PMC4525178; doi:10.1038/ncomms8866)
Supplement: Supplementary Figures and Supplementary Note — Supplementary Figures 1-18 and Supplementary Note 1 [file ncomms8866-s1.pdf]

# Supp Fig. 1

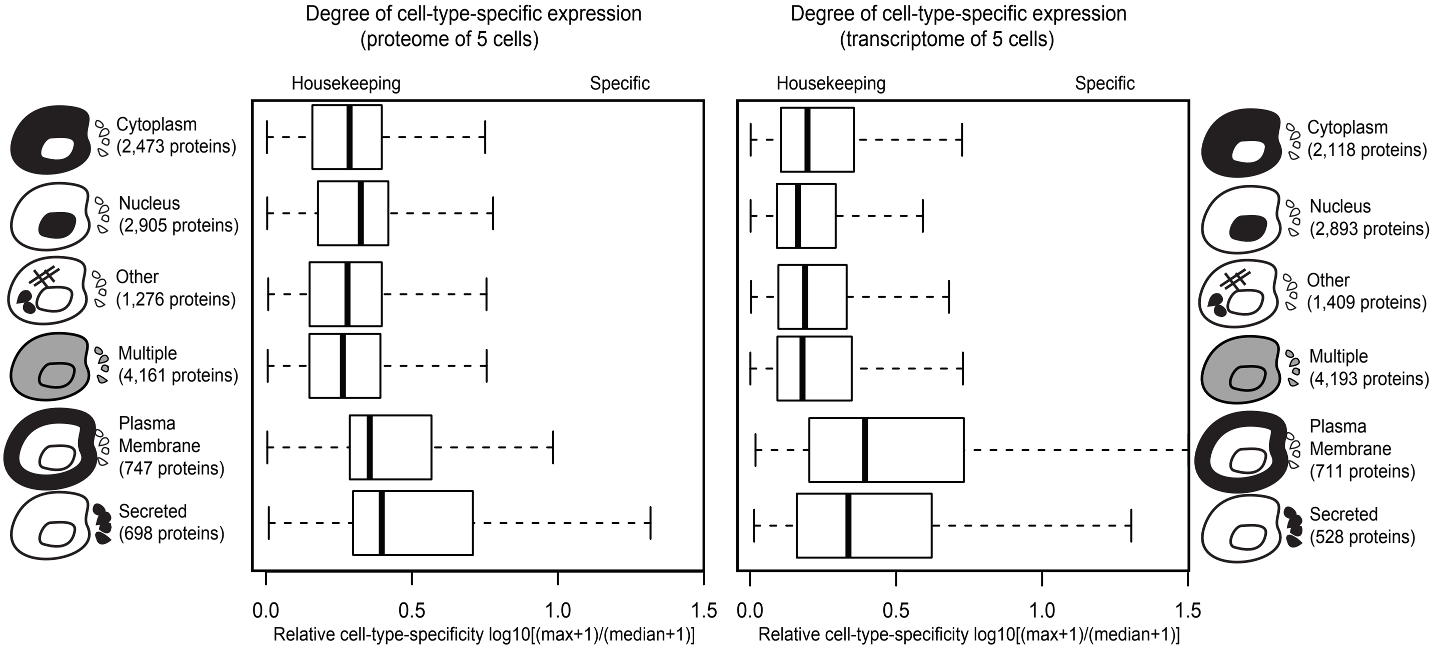


**Supp Figure 1 | Whole cell proteome data confirm that plasma membrane and secreted proteins are more cell type specific than those that localize to other cellular compartments.**
**Left** shows the numbers of proteins from each cellular compartment detected in the whole cell proteome data from Kim et al. (monocytes, CD4+ T-cells, CD8+ T cells, NK cells and B cells) and the relative cell-type-specificity. **Right** shows the same except using the CAGE data for the same 5 cell types.

# Supp. Fig. 2

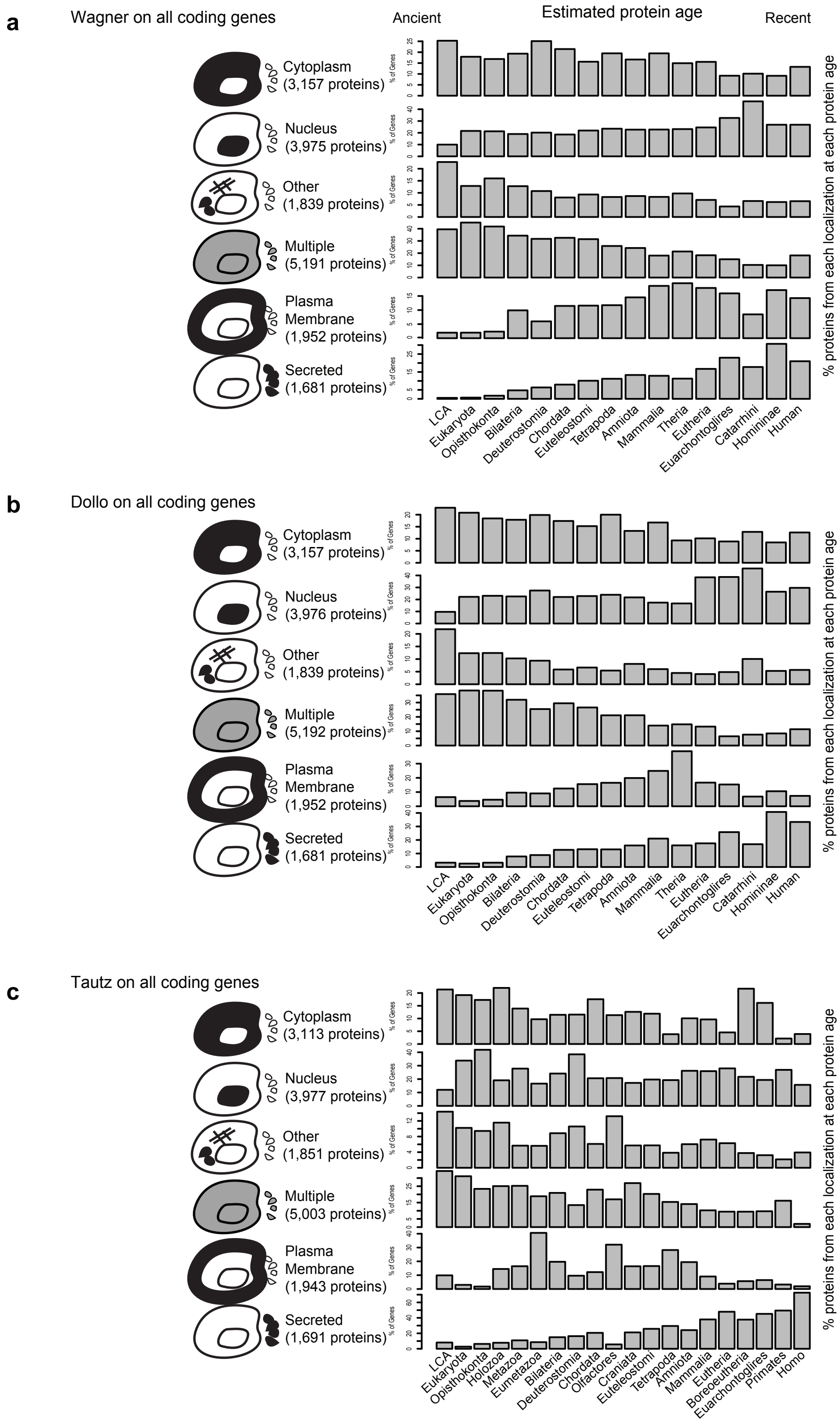


**Supp. Figure 2 | Relationship between protein subcellular localization, cell-type-specific expression and gene age.** As in **Fig. 1**, but showing age estimates using three different methods. **a.** All proteins using the methods of Wagner *et al.*, **b.** Using the methods of Dollo *et al.* and **c.** Using the methods of Tautz *et al.* Left panel in each shows the breakdown of sub-cellular localization of all human protein coding genes for which protein age estimates were available. Right panel shows the percentage of proteins within each phylostratigraphic stage targeted to a particular sub-cellular localization. Note: in all three versions, younger proteins are more likely to be localized to the plasma membrane or secreted.

# Supp. Fig. 3

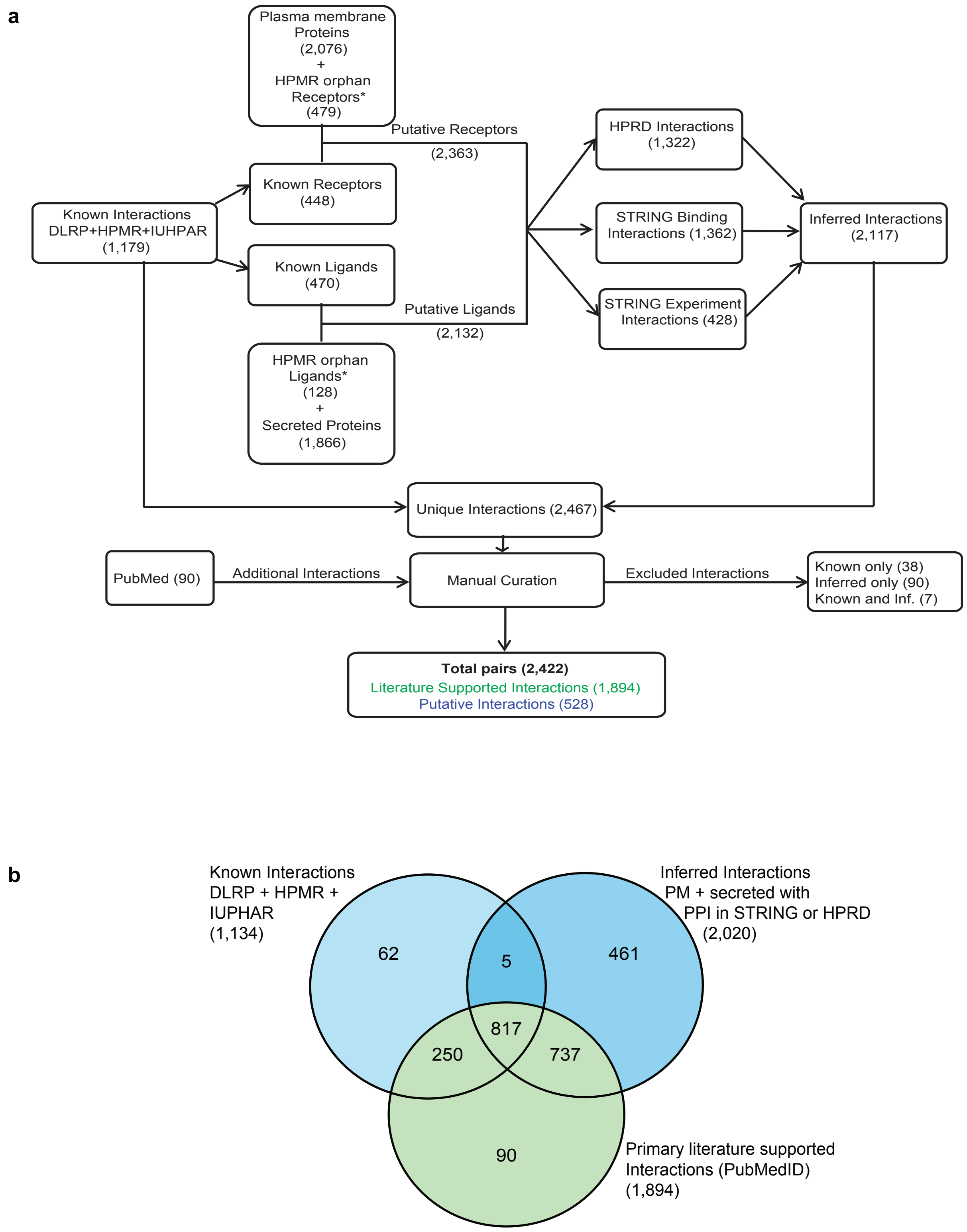


**Supp. Figure 3 | Pipeline for identifying an expanded set of ligand-receptor pairs. a.** Flow-diagram showing the incorporation of known LR pairs, and predicted LR pairs for interacting pairs of plasma membrane and secreted proteins. **b.** Break-down of primary literature supported LR pairs (green) and unsupported LR pairs (blue).

*HPMR contains orphan receptors and ligands identified as family members of receptor and ligand families, but for which no partner was known. Note: some reference interactions were deemed incorrect after manual inspection and removed. For a significant number of DLRP pairs we could find no primary literature supporting the interaction.

# Supp. Fig. 4

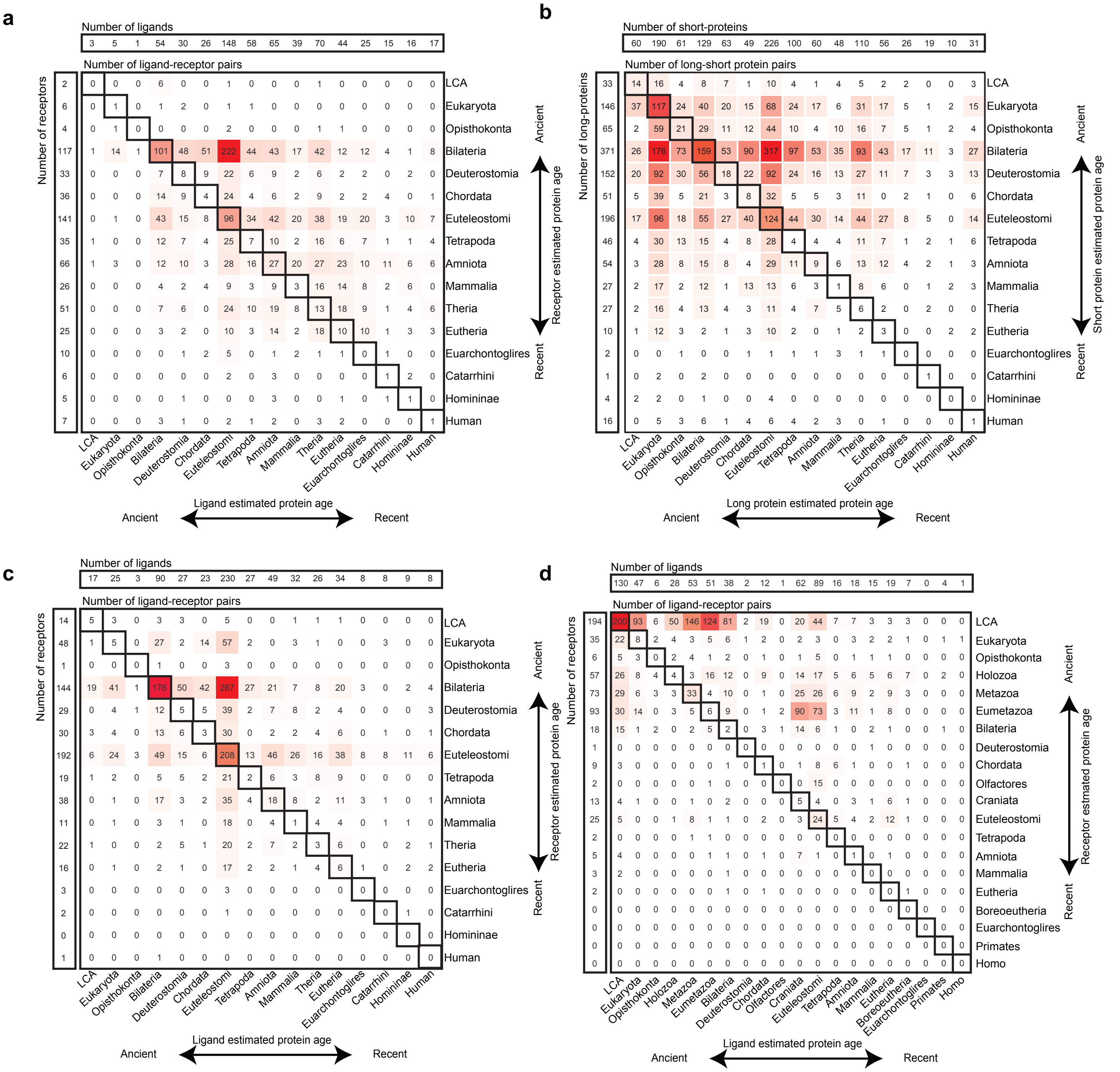


**Supp. Figure 4 | Matrices showing evolutionary period when receptors an their cognate ligands arose. a.c.d.**Show the matrices for gene age estimates according to the methods of **a.** Wagner, **c.** Dollo and **d.** Tautz. **b.** Is a control comparing gene age estimates (Wagner) of interacting random long and short proteins.

Top and left panels list the number of ligands and receptors estimated to have arisen at each phylostratum. Matrix panels show the number of ligand-receptor pairs observed between ligands of gene age X with receptors of gene age Y. Note a, is the same as **Fig. 2** and is duplicated here to aid comparison.

# Supp. Fig 5

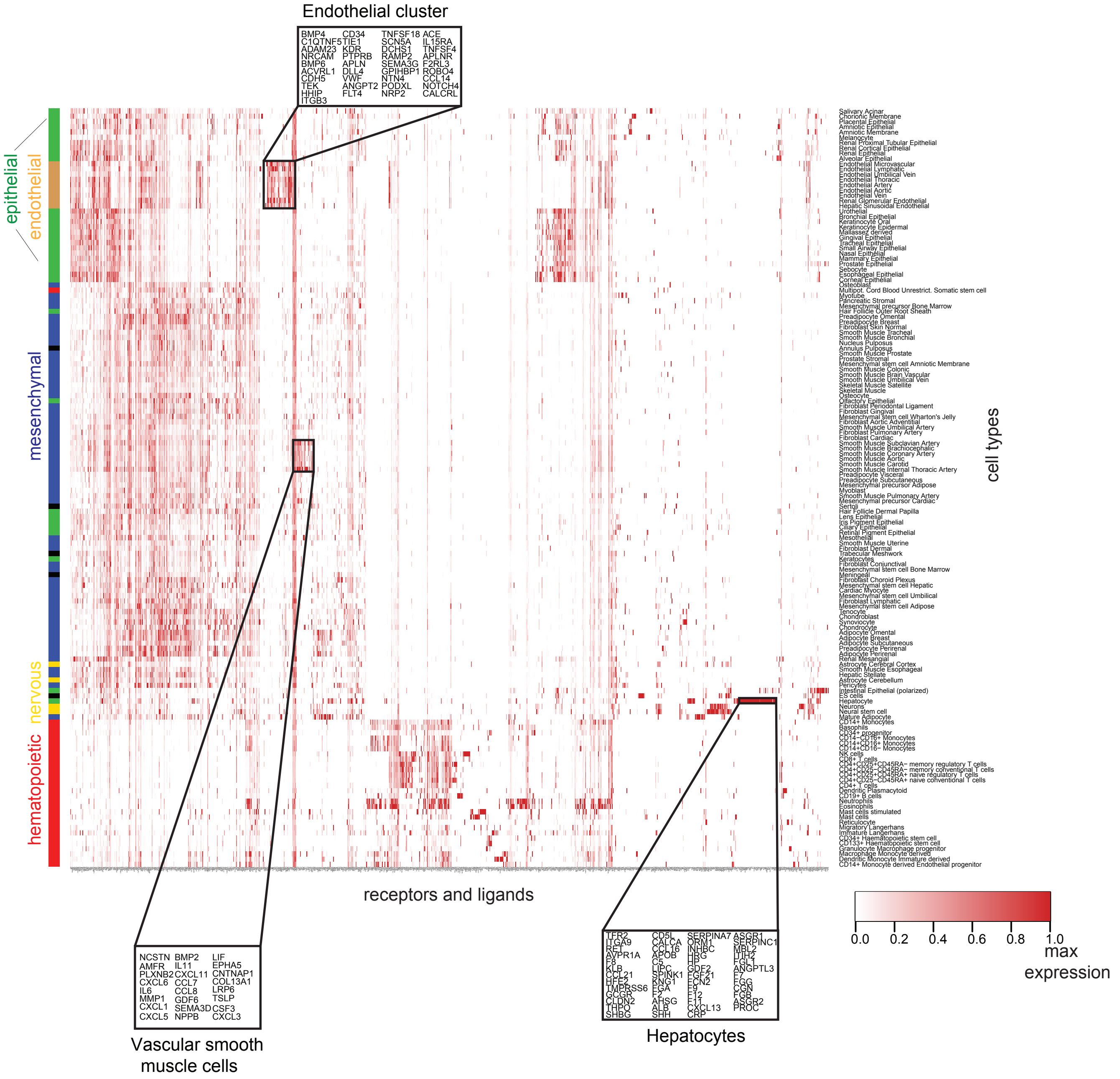


**Supplementary Figure 5 | Expression clustering by receptor/ligand and cell type.** The CAGE expression profiles of receptors and ligands detected above 10TPM are shown. For each gene the profile was normalised by dividing by the maximum expression. Thus the scale is 0-1 with red being maximum expression. The hierarchical clustering was carried in R using the hclust package and spearman correlation as similarity. The colour bars on the left correspond to cell lineages. The boxes at the top and bottom show sets of receptors and ligands that are enriched in different cell types (top - endothelial cells, bottom left - vascular smooth muscle cells, bottom right - hepatocytes).

# Supp. Fig. 6

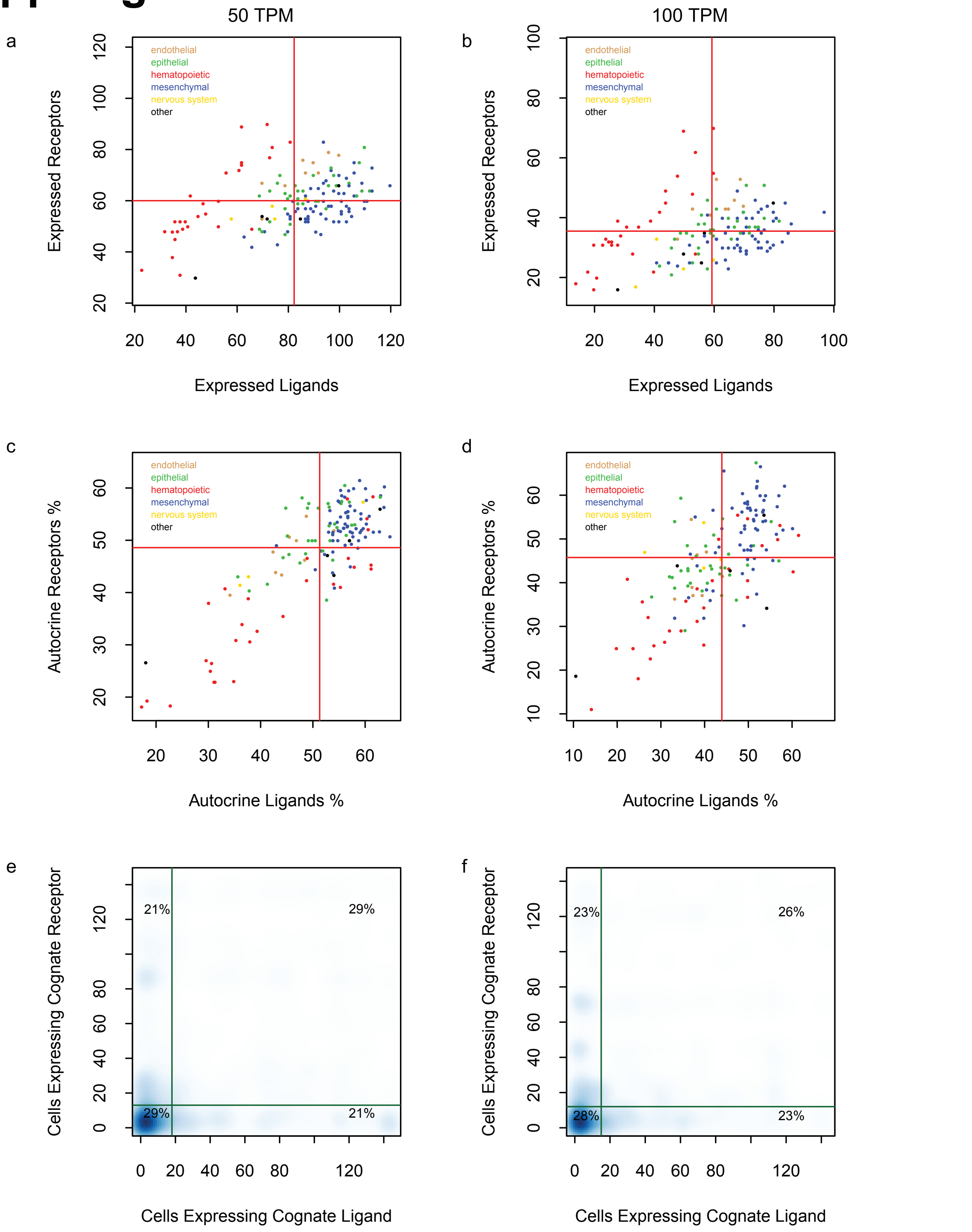


**Supp. Figure 6 | Summary statistics of receptor and ligand usage for primary cell types at 50 TPM and 100 TPM thresholds.**
**a-d.** Each data-point corresponds to a primary cell-type. Colors indicate broad lineage classes. **a.** Number of receptors vs numbers of ligands expressed in each each cell type at 50 TPM. **b** At 100 TPM. **c,d.** Autocrine signaling in primary cell types. X-axis shows fraction of ligands expressed by a given cell where the receptor is also expressed on the same cell. Y-axis shows the reciprocal for the fraction of receptors on a given cell where the ligand is also expressed, at 50TPM (**c**) and 100TPM (**d**). The red lines in a,b show the mean numbers of ligands or receptors in each plot. e. density plot showing the relative number of cells expressing a ligand and the number of cells expressing the cognate receptor at (**e**) 50 TPM or (**f**) 100 TPM. Medians shown as green lines.

# Supp. Fig. 7

a

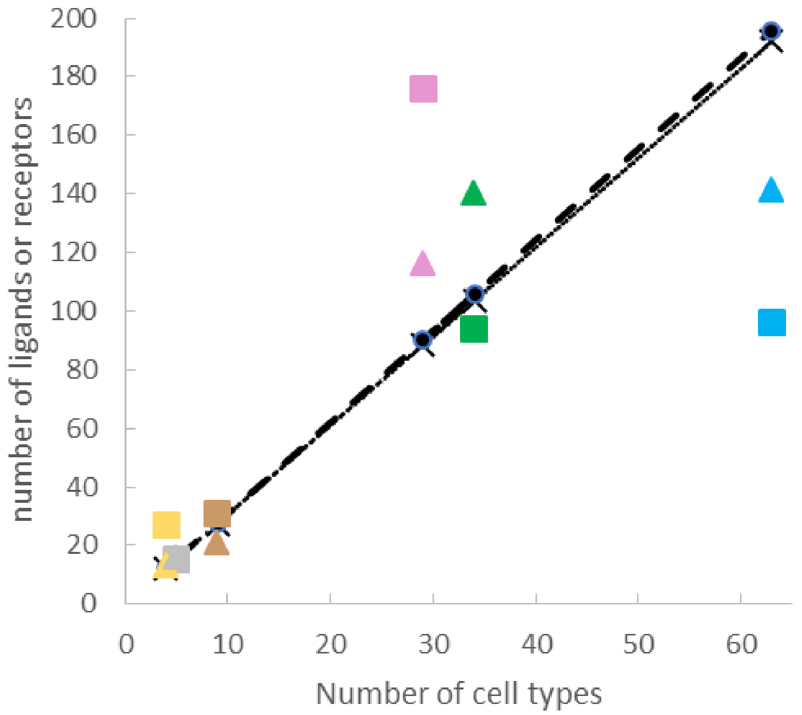


b

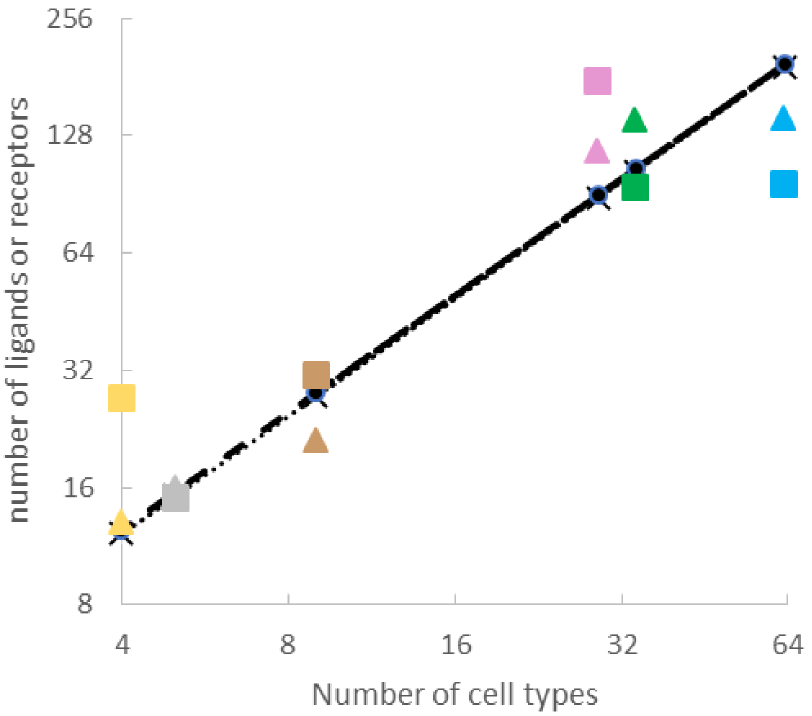


**Supplementary figure 7: Relationship between the numbers of cells assigned to each lineage and the number of observed max receivers (maximum level of a given receptor) and max transmitters (maximum level of a given ligand). a,** Dashed lines shows the expected numbers of max receivers (thin line) and transmitters (thick line) expected given the number of cells in each group. Triangles and squares show the actual observed numbers. Colours match those from Fig 5. Yellow – nervous system, grey – other, brown – endothelial cells, pink – hematopoietic cells, green – epithelial cells and blue – mesenchymal cells. Note that the hematopoietic cells have significantly more, and the mesenychymal cells have significantly fewer of the maximally expressed receptors and ligands than expected based on the number of cell types in these lineages. **b,** as in a, but plotted on log2 scale for easier viewing.

# Supp. Fig. 8

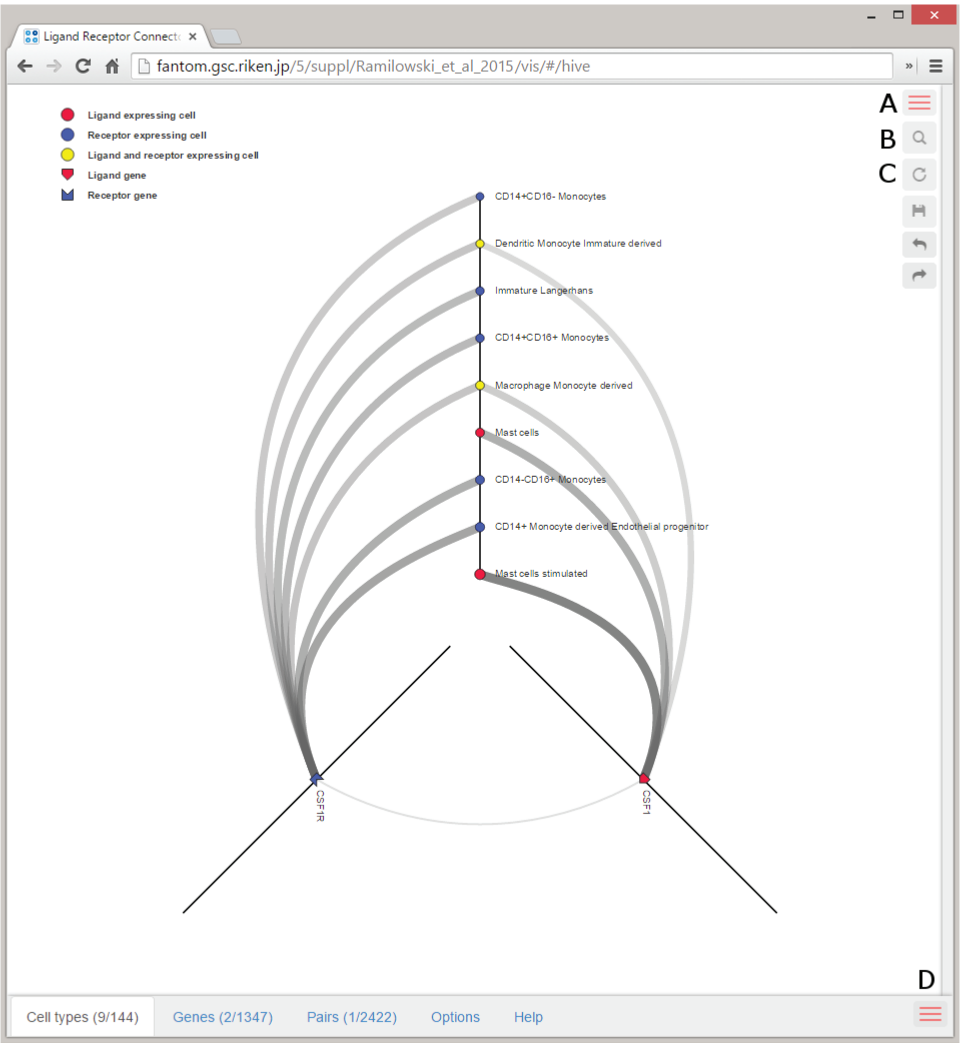


**Supp. Figure 8 | The connectome visualization interface.** **a.** toggle button used to reveal node and edge counts. **b.** Search button. **c.** toggle button used to switch between the current hive plot visualization and the force directed network visualization **d.** Toggle button used to view more information about the cell types, genes, and ligand-receptor pairs as well as control the visibility of connectome elements.

# Supp. Fig. 9

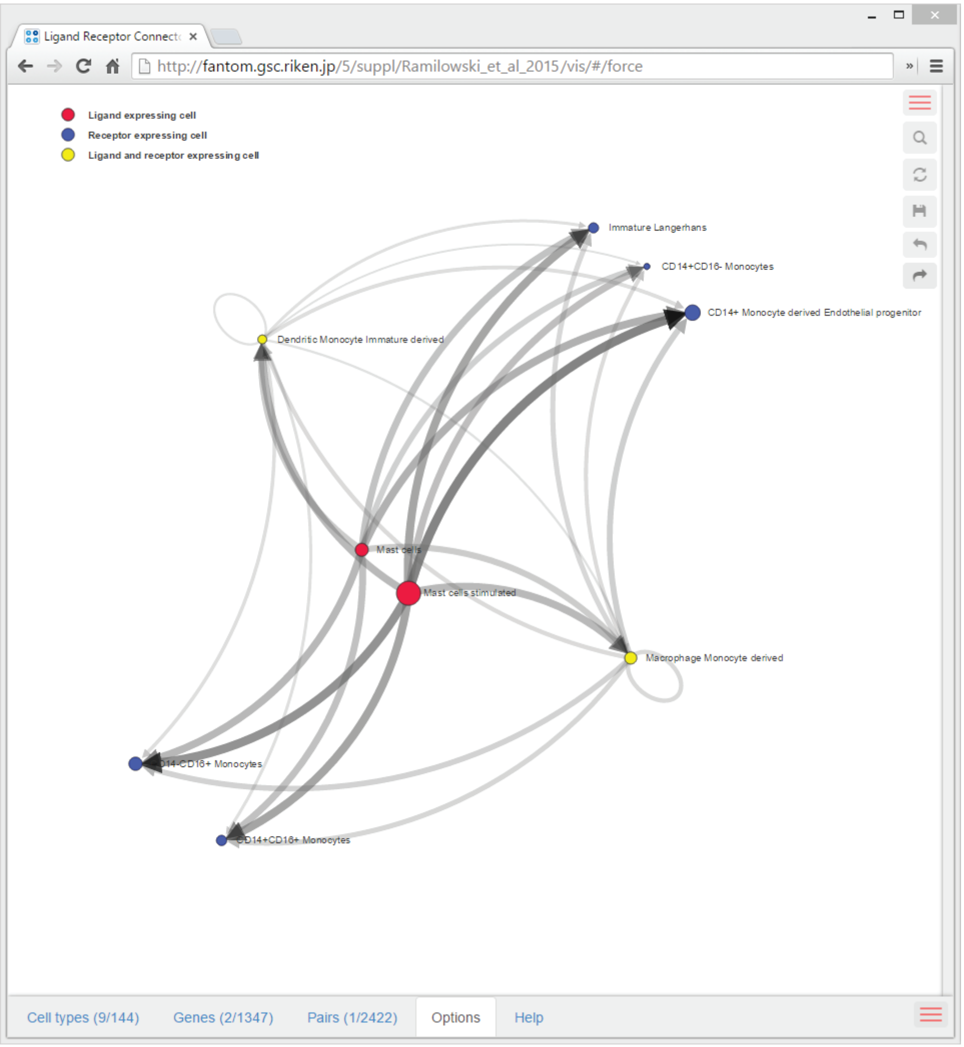


**Supp. Figure 9 | The connectome visualization interface (force directed view).**

# Supp. Fig. 10

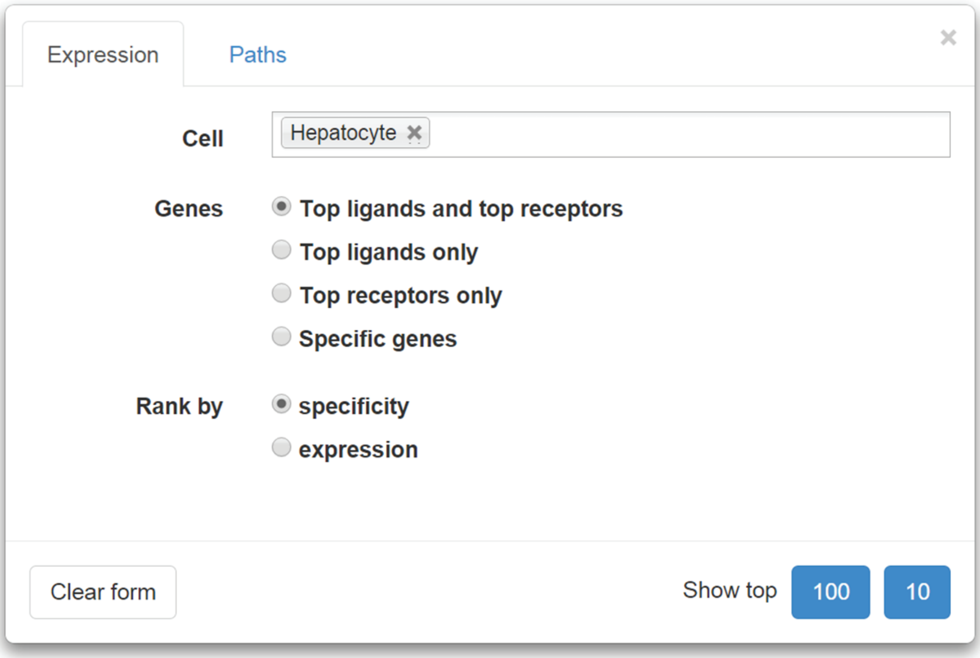


**Supp. Figure 10 | The expression search tool.** Allows user to visualize cells and genes that exhibit the top 10 expression or specificity values. Here, the search case shows the top 10 ligand genes and top 10 receptor genes expressed by hepatocytes ranked by specificity.

# Supp. Fig. 11

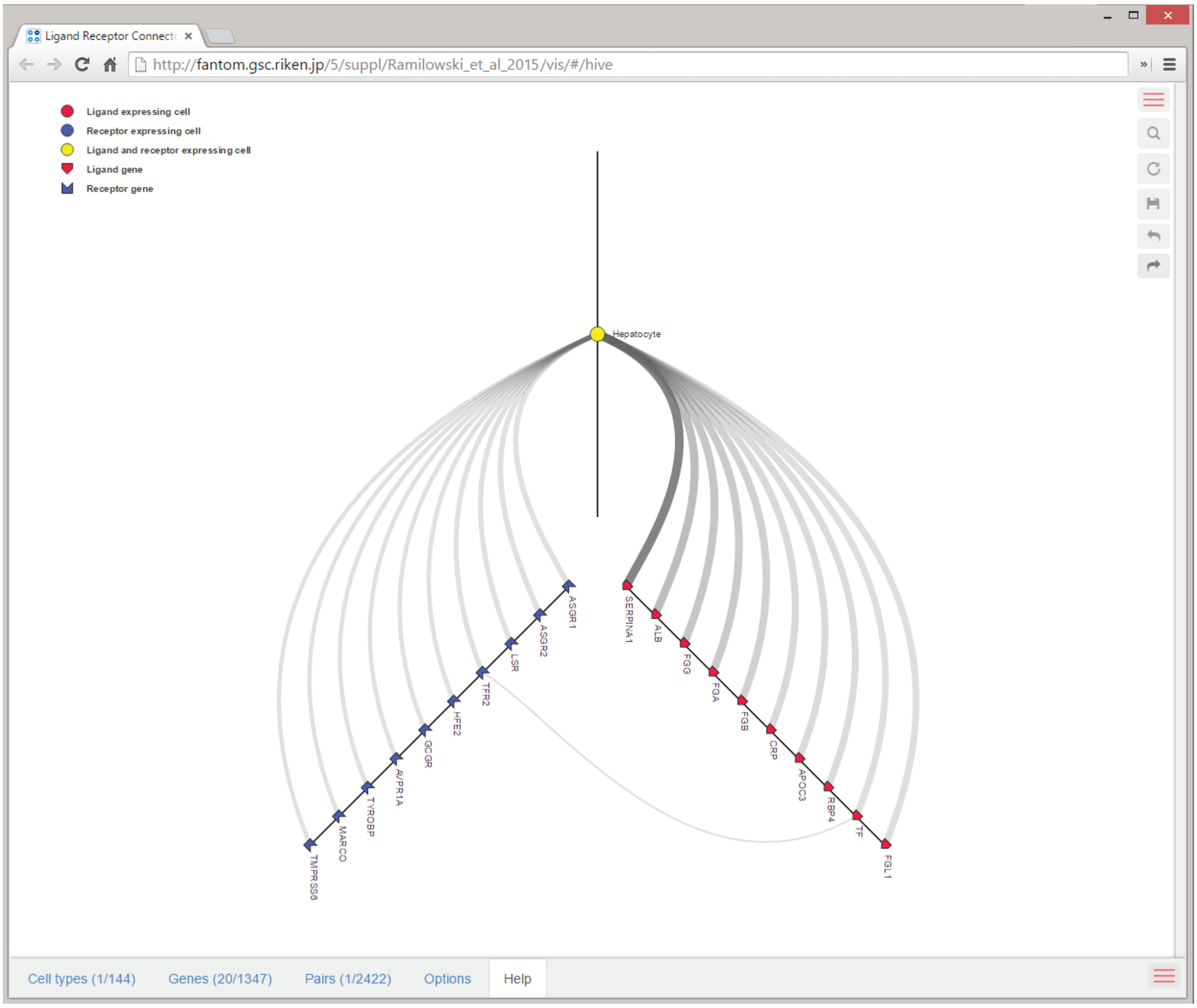


**Supp. Figure 11 | Top receptors and ligands expressed in hepatocytes.** Results of top ligands/top receptors search for hepatocytes. Shows one (1) cell type, 20 genes, and one (1) ligand receptor-pair.

# Supp. Fig. 12

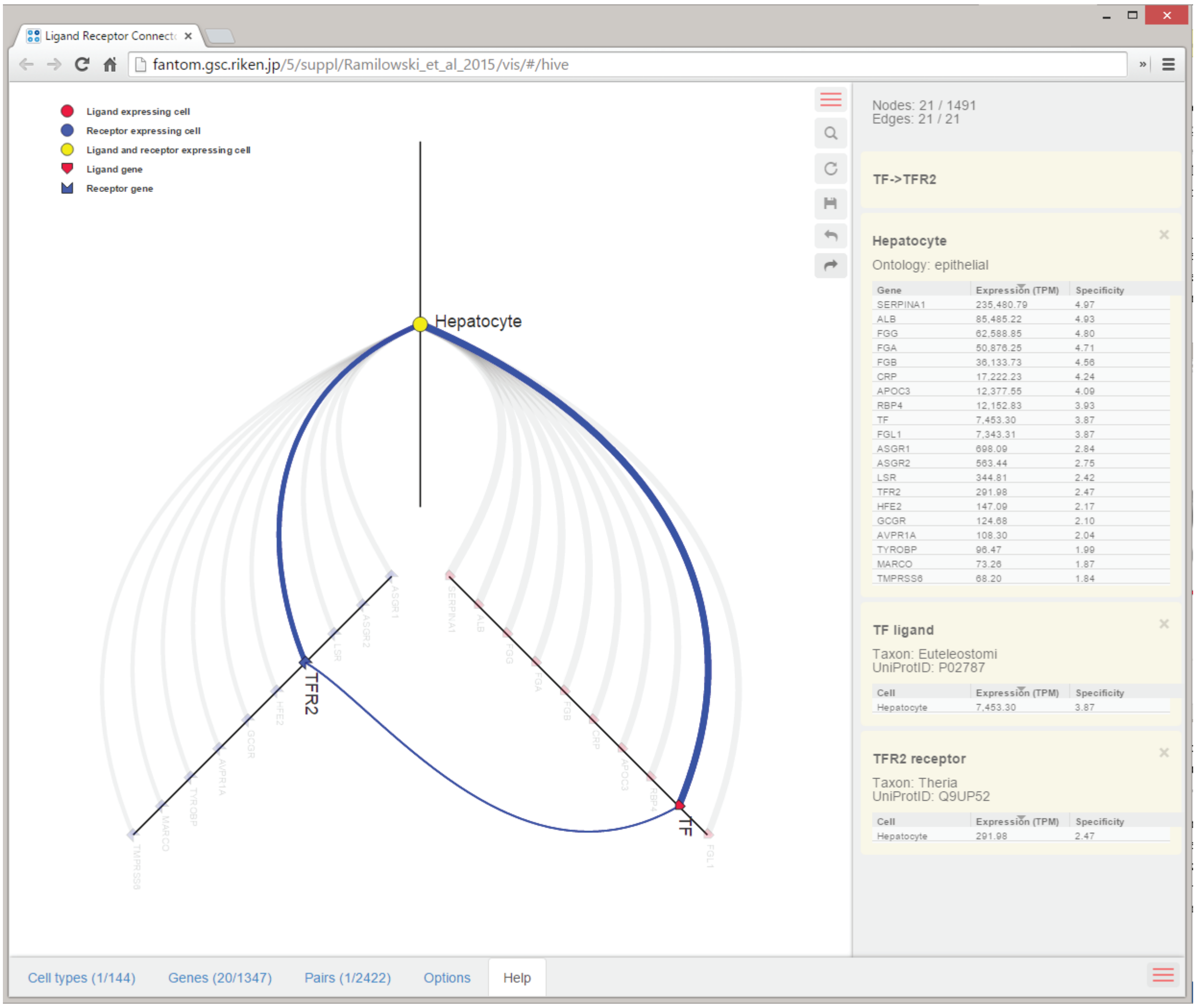


**Supp. Figure 12 | Top receptors and ligands expressed in hepatocytes - hover.** Hover or select (left click) elements in the visualization to see more information in the right panel. Here, the information on the TF ligand and TFR2 receptor in hepatocytes is displayed. The hepatocyte-TF-TFR2-hepatocyte path is highlighted in blue.

# Supp. Fig. 13

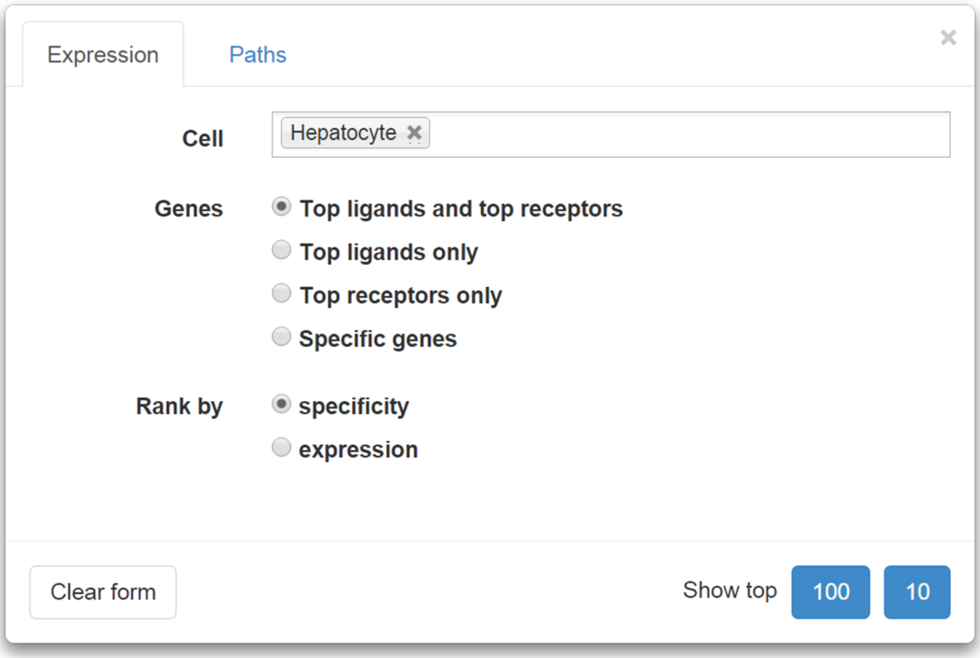


**Supp. Figure 13 | The pathway search tool.** Allows user to visualize cells and ligand-receptor pairs that participate in the top 10 cell-cell communication paths ranked by specificity sum or expression product.

# Supp. Fig. 14

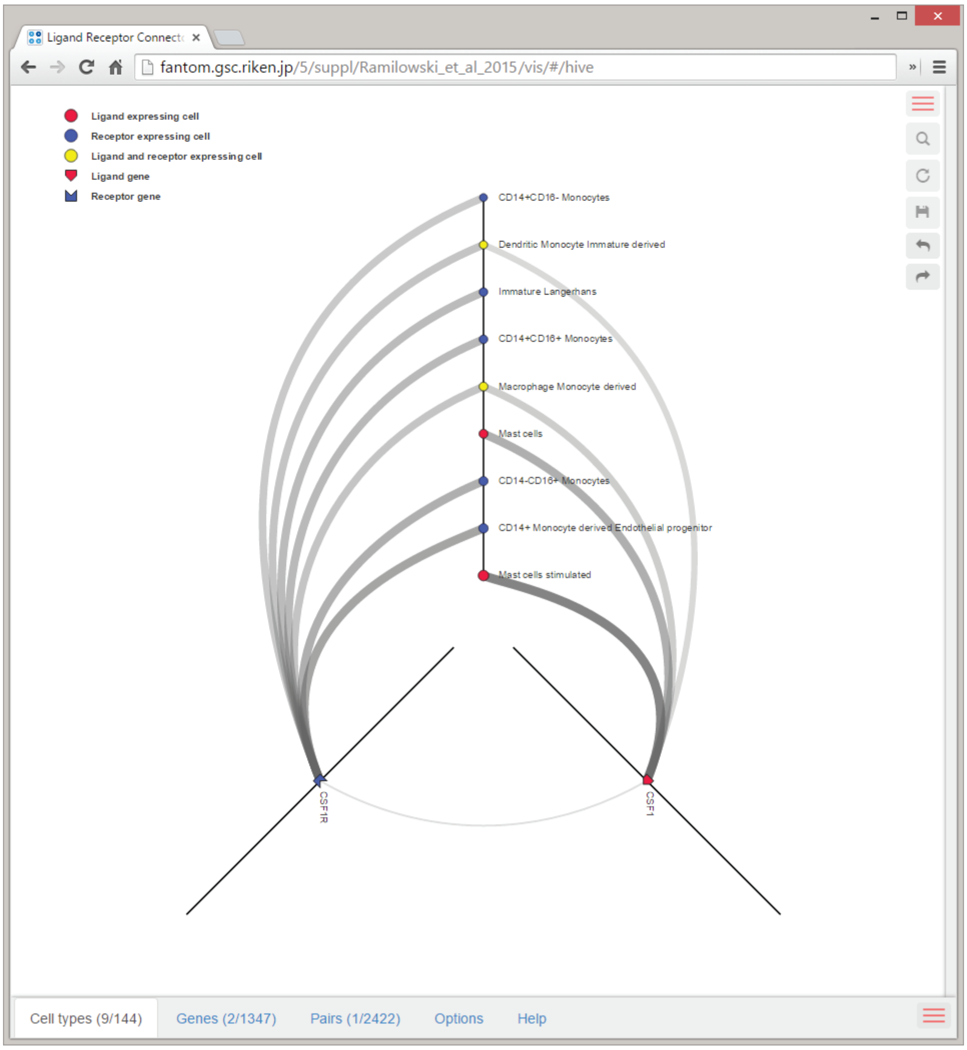


**Supp. Figure 14 | Results of using the paths search tool to find cell types communicating through the CSF1-CSF1R ligand-receptor pair.**

# Supp. Fig. 15

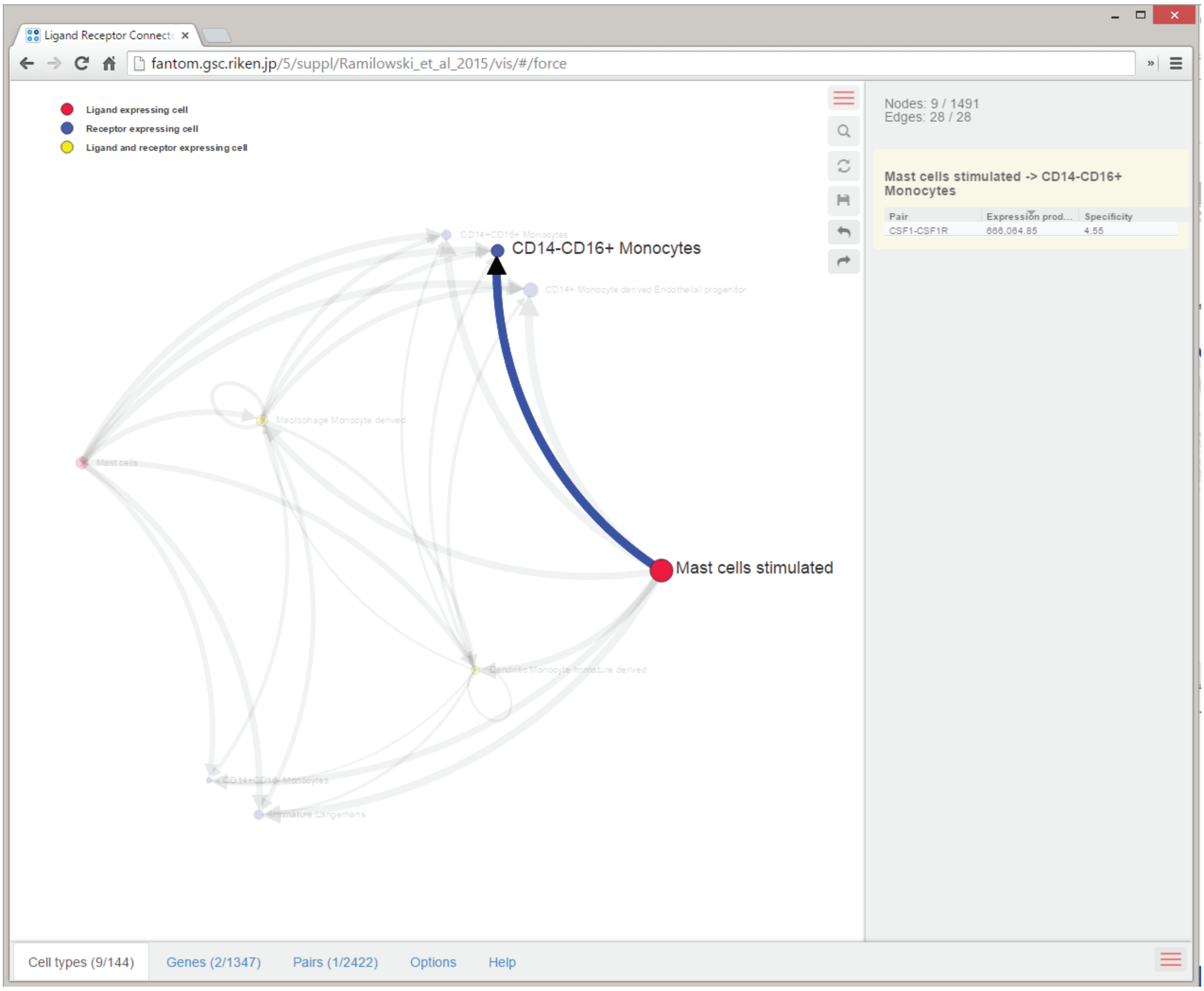


**Supp. Figure 15 | Results of using the paths search tool to find cell types communicating through the CSF1-CSF1R ligand-receptor pair - force directed view.** Hover over cell-cell edges to get expression product and specificity sum values.

# Supp. Fig. 16

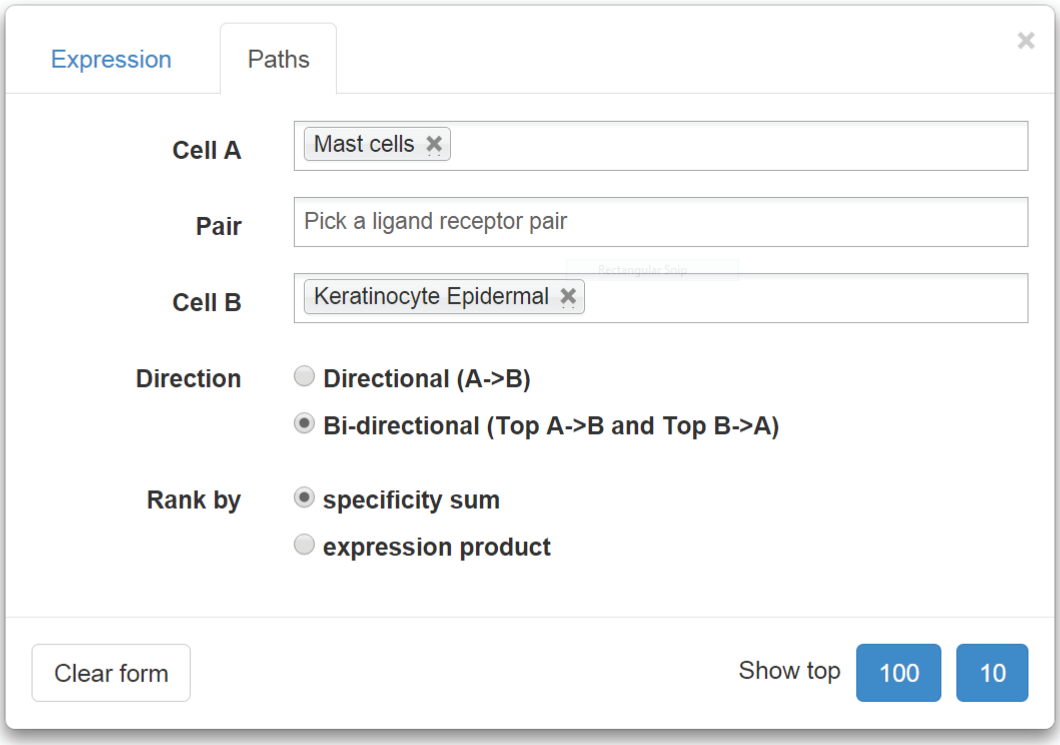


**Supp. Figure 16 | The pathway search tool2.** Allows user to visualize cells and ligand-receptor pairs that participate in the top 10 cell-cell communication paths between mast cells and epidermal keratinocytes, ranked by specificity sum.

# Supp. Fig. 17

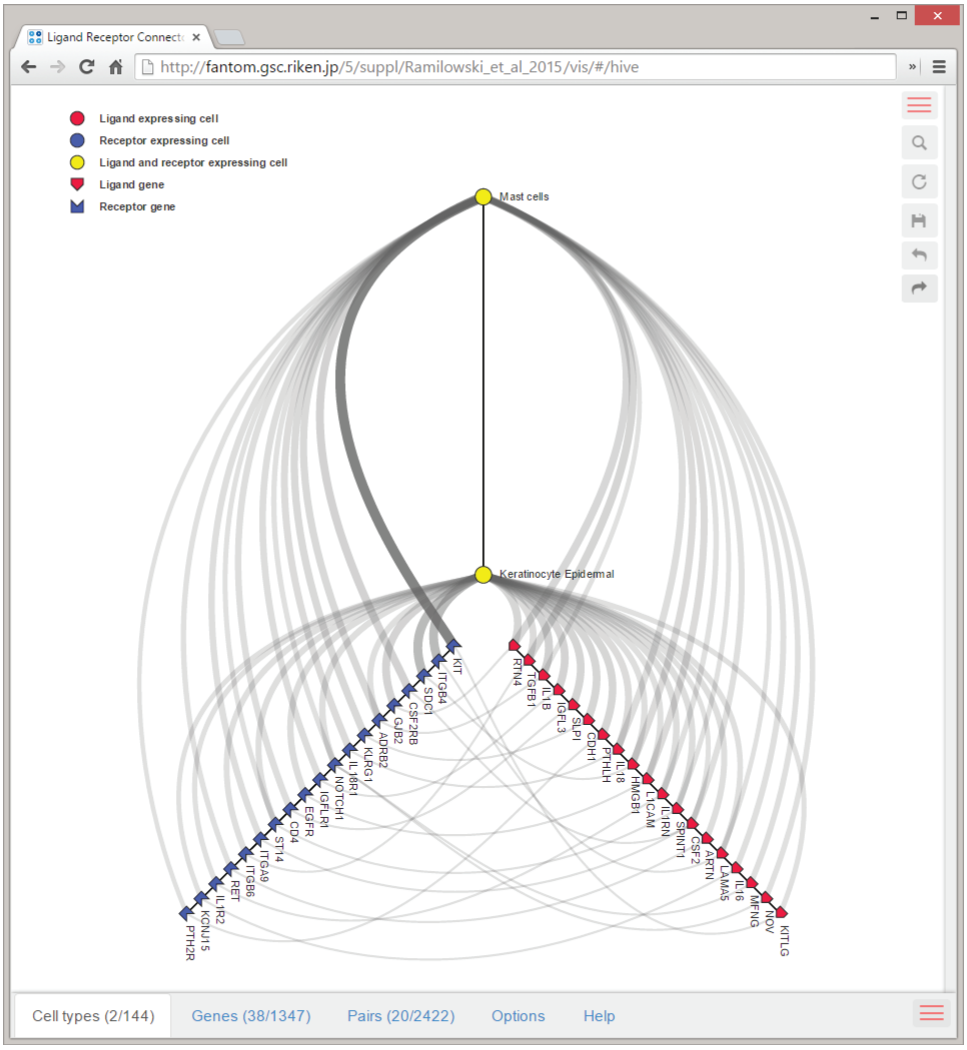


**Supp. Figure 17 | Results of using the paths search tool to find the top ligands and receptors used for communication between mast cells and epidermal keratinocytes.** Results of using the paths search tool to find elements involved in the top communication paths between mast cells and epidermal keratinocytes. R esults include two cell types, 38 genes, and 20 ligand receptor-pairs.

# Supp. Fig. 18

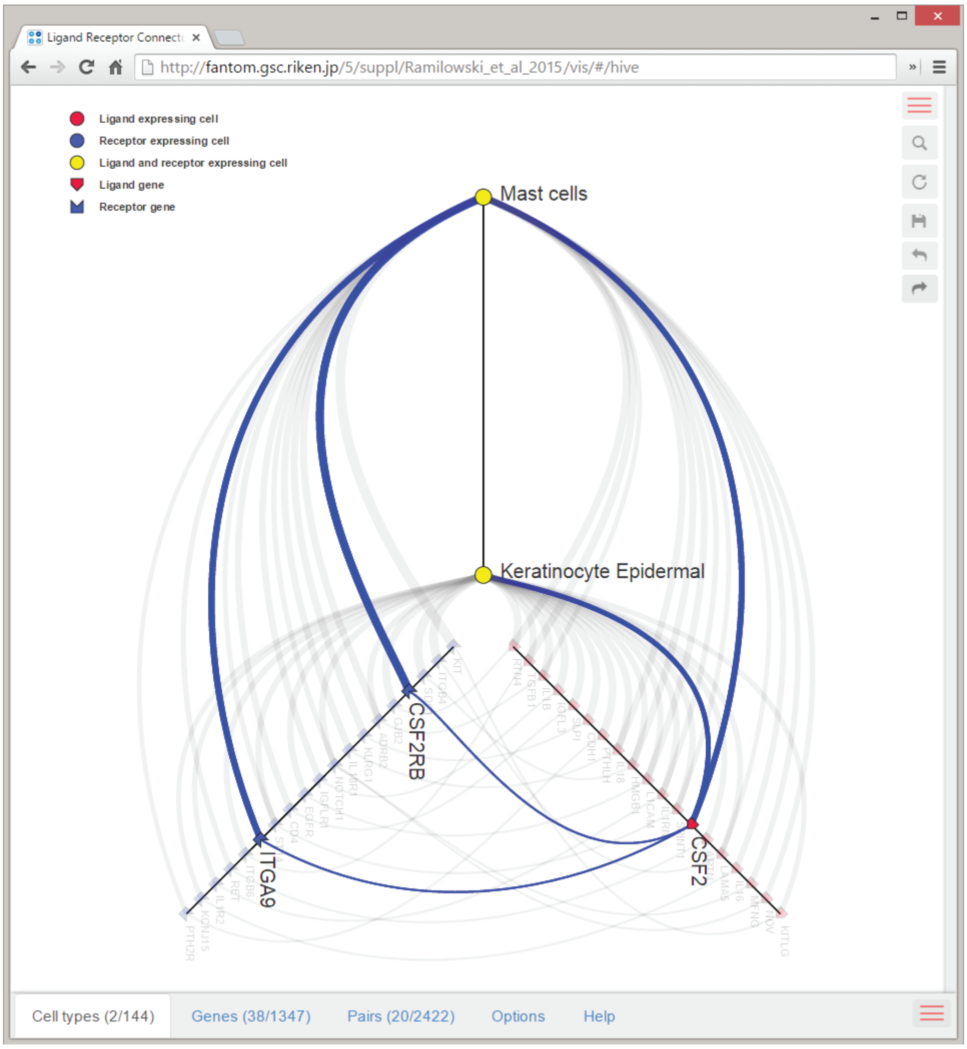


**Supp. Figure 18 | Results of using the paths search tool to find the top ligands and receptors used for communication between mast cells and epidermal keratinocytes.** As in Supp fig 17 but shows highlighted paths after hovering over CSF2.

**Supplementary Note 1: Use case studies for the webtool. Examining ligand-receptor pairs and communication between primary cell types.**

## Introduction

In the accompanying paper of Ramilowski et al. '*A draft network of ligand-receptor mediated multicellular signaling in human*' we present the first large-scale map of cell-to-cell communication, examining signaling between 144 human primary cell types using 2,422 putative and literature supported ligand-receptor pairs. With up to hundreds of potential interactions between any two of these 144 primary cell types, there are millions of possible cell-cell communication paths across the entire network. Static visualization of such complex networks not only can be obscure and impractical but also difficult. With that, and to benefit the research community, we provide an online resource that visualizes, on demand, our cell-cell communication network for any given subset of the ligand-receptor pairs and profiled primary cells. The interface is available at: http://fantom.gsc.riken.jp/5/suppl/Ramilowski_et_al_2015/. This short note briefly presents a few selected study cases to help users gain a more intuitive understanding of the visualization tool (a more comprehensive guide on how to use the tool is given in the online 'Help' menu).

The main interface area of the tool (**Supplementary Figure 8**) starts with a 'hive' view of the CSF1-CSF1R ligand-receptor pair and the cells that most strongly communicate via the pair. In this view, primary cell types are shown as circular nodes along the vertical axis while ligand and receptor genes are shown, as rectangular nodes along the right and left angled axes, respectively. The node color differentiates between ligand-expressing cells (red), receptor-expressing cells (blue), and cells expressing both ligands and receptors (yellow); the genes are indicated by different color and shape: ligands (red irregular convex pentagons) and receptors (blue irregular concave pentagons). Edges are shown both for the gene-cell expression associations and ligand-receptor pairs. The ligand-receptor expression edges are weighed by expression value. Additional information on currently visualized network etc., can be accessed through various toggle buttons as we further describe.

With the toggle button in the upper right of the interface (labeled **A**) a user can view node and edge counts. Hovering over, or selecting elements in the visualization, gives additional information on visualized nodes and edges.

The type and number of currently visible elements can be seen in the information bar below the visualization. By pressing the toggle button (labeled **D**) the user can view more information about the cell types, genes, and ligand-receptor pairs as well as control the visibility of connectome elements. In the options tab a user can change, independently, the ligand and receptor expression TPM cutoff (defaults set to 10 TPM) as well as toggle the label visibility.

Using the search button (labeled **B**) a user can find and visualize specific expression edges and/or ligand-receptor pair edges. More details on this, and other features, are given in example use cases described below.

The toggle button labeled **C** is used to switch between the hive plot visualization and the force directed network visualization (**Supplementary Figure 9**). In the force directed visualization only the cell types are shown with edges representing the sum of all communication paths between them. Edges are weighed by the ligand-receptor expression products summed over gene pairs. In this view it is useful to set the expression edge cutoff filter in the options panel.

**Use case 1: What are the most specific ligands and receptors expressed by a given cell? (Example of Hepatocytes)**

Press the find button (labeled **B** in **Supplementary Figure 9**). The search dialog box will appear. The first tab is the expression search tool. In the “Cell” text box type “hep” without quotes. You should see a dropdown list of all cell types that contain the text “hep” (**Supplementary Figure 10**). Select “Hepatocyte”. Leave the genes input as “Top ligands and top receptors” to indicate you are searching for the top expression values in each gene class. Leave the “Rank by” selection as “specificity” to show top expression edges ranked by cell specificity. The specificity is defined as $\log_{10}(E_{i,j} + 1) - \log_{10}(M_i + 1)$ where $Ei,j$ is the expression of gene $i$ in cell $j$ and $M_i$ is the median expression of gene $i$ across all cells. Click “Show top 10”. (Note that choosing rank by ‘expression’ would show the top expressed, other than most specific, ligands/receptors in hepatocytes)

The entities bar across the bottom (**Supplementary Figure 11**), shows the 10 most specific ligands (along the rightward sloped axis) and the 10 most specific receptors, (along the leftward sloped axis) for ‘Hepatocyte’ (shown as a circular node on the vertical axis). If you do not see a hive plot, verify you are in the hive visualization mode (labeled **C** in **Supplementary Figure 8**). Edges connecting the cell node and genes are expression edges weighted by their expression value. You will also see that the search tool found one ligand-receptor pair between visible genes shown as an edge between the TF ligand gene and the TFR2 receptor gene. Additional information about nodes and edges, including expression and specificity values, can be found by opening the right side information panel (labeled **A** in **Supplementary Figure 8**).

Hover over nodes or edges for more information. Left click to select individual elements to display them in the information panel. Shift left-click on the TFR2 receptor gene node to select the entire Hepatocyte-TF-TFR2-Hepatocyte communication path. Hover over the Hepatocyte to TF edge to see the expression value and specificity value for the TF gene in Hepatocytes (see **Supplementary Figure 12**).

**Use case 2: which cells are communicating the most via selected ligand-receptor pair(s)? (Example of CSF1-CSF1R ligand-receptor pair)**

Press the search button. Navigate to the “Paths” tab. In the “Pair” text box type “CSF1” (without quotes). You will see a list of all ligand-receptor pairs containing CSF1 ligand or CSF1R receptor. Click “CSF1-CSF1R”. Note that in this case direction and ranking are not relevant and will return the same results. Click “Show top 10” (**Supplementary figure 13**).

The entities bar will indicate that nine cell types are now visible (see **Supplementary figure 14**). These are the cells involved in the top 10 cell-cell communication paths via the CSF1-CSF1R pair. Note that the hive visualization will show all expression edges between visible cell types and genes even if only a subset of ligand and receptor expression combinations make up the top ten communication paths.

Switch to the force directed visualization (button labeled **C** in **Supplementary figure 8**) and hover over expression edges to display the expression product and specificity sum for a given cell-cell pair (see **Supplementary figure 15**).

**Use case 3: what are the top 10 paths used to communicate between any given two cells? (Example of mast cells and epidermal keratinocytes)**

Ensure you have returned to the hive visualization (labeled **C** in **Supplementary figure 8**). Press the search button. Navigate to the “Paths” tab. Press “Clear form” if you have any information left from previous searches. In the “Cell A” text box type “mast” and select “Mast cells”. In the “Cell B” text box type “ker” and select “Keratinocyte Epidermal”. Leave the direction option set to “Bi-directional” to find genes participating in the top 10 cell-cell communication paths in each direction. Leave the “Rank by” option set to “specificity sum” to rank communication paths by the sum of ligand and receptor specificities for a given ligand-cell/receptor-cell pair. Click “Show top 10” (**Supplementary figure 16**).

The entities bar will show two (2) cell types, 38 genes, and 20 ligand receptor pairs (see **Supplementary figure 17**).

This visualization shows genes participating in the top communication paths between mast cells and epidermal keratinocytes in each direction. Note again that the visualization will show all expression edges between visible cell types and genes even if a given expression value is not part of the top communication paths. Hover over the CSF2 ligand to highlight communication paths involving this gene (see **Supplementary figure 18**).

**Queries on interface or content please email:**

Interface developed by Jayson Harshbarger <jayson.harshbarger@riken.jp>
Content maintained by Jordan Ramilowski <jordan.ramilowski@riken.jp>
